# Multimodal optoacoustic imaging: methods and contrast materials

**DOI:** 10.1039/d3cs00565h

**Published:** 2024-05-13

**Authors:** Zhenyue Chen, Irmak Gezginer, Quanyu Zhou, Lin Tang, Xosé Luís Deán-Ben, Daniel Razansky

**Affiliations:** a Institute for Biomedical Engineering and Institute of Pharmacology and Toxicology, Faculty of Medicine, University of Zurich Switzerland daniel.razansky@uzh.ch; b Institute for Biomedical Engineering, Department of Information Technology and Electrical Engineering ETH Zurich Switzerland

## Abstract

Optoacoustic (OA) imaging offers powerful capabilities for interrogating biological tissues with rich optical absorption contrast while maintaining high spatial resolution for deep tissue observations. The spectrally distinct absorption of visible and near-infrared photons by endogenous tissue chromophores facilitates extraction of diverse anatomic, functional, molecular, and metabolic information from living tissues across various scales, from organelles and cells to whole organs and organisms. The primarily blood-related contrast and limited penetration depth of OA imaging have fostered the development of multimodal approaches to fully exploit the unique advantages and complementarity of the method. We review the recent hybridization efforts, including multimodal combinations of OA with ultrasound, fluorescence, optical coherence tomography, Raman scattering microscopy and magnetic resonance imaging as well as ionizing methods, such as X-ray computed tomography, single-photon-emission computed tomography and positron emission tomography. Considering that most molecules absorb light across a broad range of the electromagnetic spectrum, the OA interrogations can be extended to a large number of exogenously administered small molecules, particulate agents, and genetically encoded labels. This unique property further makes contrast moieties used in other imaging modalities amenable for OA sensing.

## Abstract 2

Combining optoacoustics with other imaging techniques helps bring its powerful label-free functional readings and molecular sensitivity into precise anatomical context while providing an essential cross-validation against established imaging methods.

## Graphical abstract

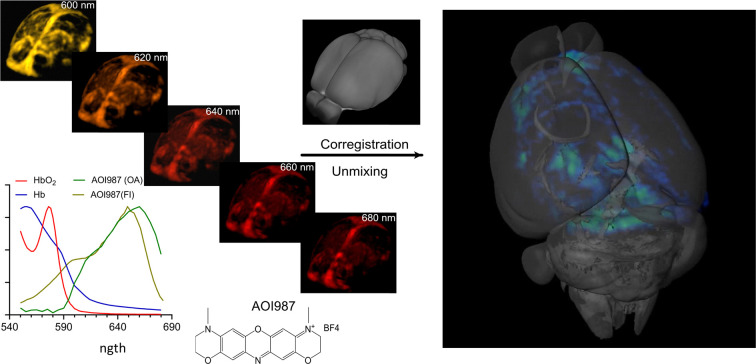

## Introduction

**Fig. 1:**
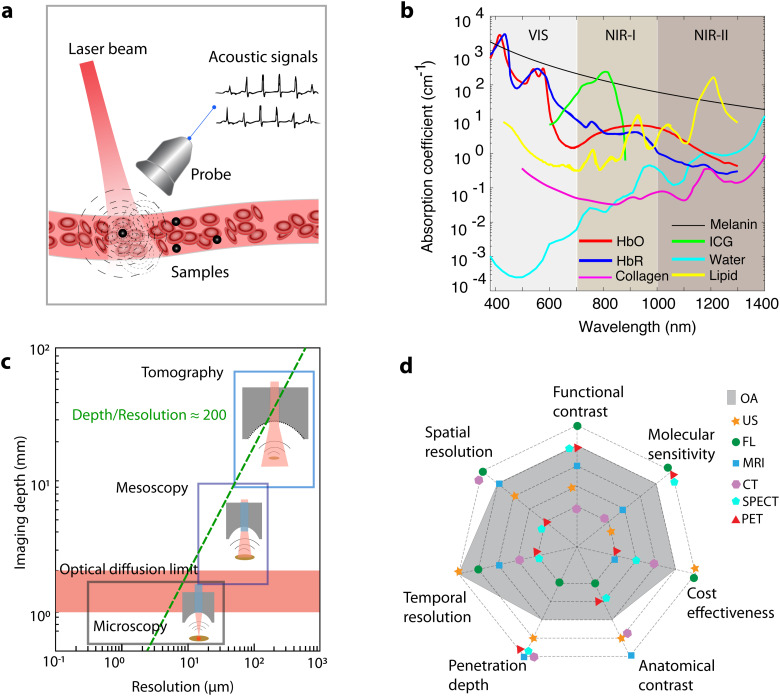
Optoacoustic (OA) imaging and the rationale of combining OA with other bioimaging modalities. (a) Schematic drawing of the working principle underlying OA imaging. (b) Absorption coefficients of major endogenous tissue chromophores at physiologically relevant concentrations as well as representative exogenous contrast agent indocyanine green (ICG). HbO, HbR, melanin (skin), water, lipid and ICG data adapted from https://omlc.org/spectra/. Collagen spectrum from ref. 12. (c) The multi-scale nature of OA imaging, covering studies from the microscopic to macroscopic levels. (d) Performance envelope of OA imaging in comparison to other modalities, underpinning its complementary advantages and the need for multi-modality integration.

Optoacoustic (OA) imaging, also known as photoacoustic imaging, synergistically combines optical excitation and ultrasound (US) detection to retrieve comprehensive anatomical, functional, metabolic, and molecular information from biological tissues. The physical principle underlying the technique entails illumination of the tissue of interest with short light pulses, typically of nanosecond duration, followed by thermoelastic expansion and detection of acoustic radiation generated *via* transient light absorption by tissue chromophores (Fig. 1(a)). While the underlying photophonic phenomenon was first described in 1880,^1^ rapid development of biomedical applications only commenced at the turn of the 21st century.^2,3^ OA imaging translates the molecular specificity of light into deep-seated areas by capitalizing on the low scattering of US to break through the depth barriers imposed by light diffusion in living tissues.^4^ The versatile contrast of OA imaging, which is based on optical absorption, allows for sensing and spatially resolving spectrally-distinctive endogenous chromophores essential to biological function, such as hemoglobin in its oxygenated (HbO) and deoxygenated (HbR) forms, melanin, water, collagen, lipids and others (Fig. 1(b)). Due to the strong intrinsic hemoglobin contrast, OA represents a valuable tool to study the evolution of important hallmarks of cancer such as angiogenesis, hypoxia and hypermetabolism.^5,6^ Furthermore, considering that most substances absorb light across a broad range of the electromagnetic spectrum, the OA interrogations can be extended to a large number of exogenously administered small molecules, particulate agents, as well as targeted, activatable, and genetically-encoded labels.^7–11^

Following two decades of rapid technological developments, state-of-the-art OA embodiments can visualize life at multiple spatial scales, ranging from subcellular structures to entire organs, with the same type of optical absorption contrast (Fig. 1(c)).^13,14^ Optical-resolution OA microscopy with focused excitation light covers depths within the transport mean free path of photons (∼1 mm in biological tissues),^10^ thus facilitating capillary-level microvascular imaging with a typical resolution of a few microns, which can further be refined to cover sub-cellular structures.^15^ OA mesoscopy uses instead unfocused illumination and broadband focused US detection to overcome the hard penetration-resolution trade-offs in optical imaging, hence enabling high resolution imaging of deep tissues in the diffuse regime of light.^7,8^ Tomographic image acquisition and rendering with multi-element transducer arrays further enable a real-time imaging capability essential for capturing rapid biological dynamics, such as cardiac or neural activity, at the whole-organ level in mice.^16,17^ These unique advantages have attracted growing attention within the biomedical research community, further fostering the development of new theranostic chemical agents.^18^

Despite its powerful technical capabilities, OA suffers from several drawbacks, including limited soft tissue contrast and penetration depth, inevitable trade-offs between penetration and spatial resolution, as well as limited sensitivity and specificity (Fig. 1(d)). More importantly, OA contrast primarily stems from optical absorption, rendering it less sensitive to other contrast mechanisms related to scattering, fluorescence, acoustic, and magnetic properties of tissue. This motivated the combination of OA with other imaging modalities to fully exploit the unique advantages and complementarity of the method toward a more comprehensive understanding of biological processes.

Due to the inherent hybrid optical and acoustic nature of OA, multi-modality efforts have mainly been focused on hybridization with US,^19–23^ fluorescence (FL),^24–29^ and other optical imaging modalities.^30–38^ Recent progress on multimodal OA has also seen promising combinations with magnetic resonance imaging (MRI),^39–46^ X-ray computed tomography (CT),^14,47,48^ single-photon-emission computed tomography (SPECT),^47,49^ or positron emission tomography (PET)^50–53^ to exploit entirely different types of anatomical, functional, and molecular contrast. Multimodal imaging expands the contrast dimension to aid a comprehensive understanding of biological structure and function, at the expense of a more complex design of imaging systems and contrast materials.^18,25,39,46,54–60^ This review delves into hybridization efforts of OA with other imaging modalities, development of multimodal contrast materials, as well as emerging applications in preclinical research and clinical diagnostics.

## Merging optoacoustic imaging with ultrasound

OA and US imaging share common advantages, such as the use of non-ionizing radiation, real-time imaging capability, high spatio-temporal resolution, and portability.^61^ Both techniques are based on acquisition of time-resolved pressure signals, which facilitates hybridization. While US renders important structural and blood flow contrast, OA provides additional oxygenation and molecular readings both in preclinical and clinical applications.^22^ Moreover, the dual-modality combination enables more accurate probing into heterogeneous acoustic and optical properties of living tissues, thus improving image reconstruction performance, data quantification and interpretation.^23,62,63^ The synergistic combination with the well-established US technique can further expedite the clinical adoption of the OA technology.

### OA–US imaging systems

High-resolution US and OA imaging is based on point-by-point mechanical scanning of a focused transducer *e.g. via* motorized translational stages^21,64,65^ or a slider-crank scanner.^66^ In multimodal OA–US systems, data acquisition is sequentially switched between the OA and US modes. Anatomical US images were reported to extract the contour maps of uneven surfaces to guide dynamic focusing.^21^ The collected US echoes have also been used to remove the multi-reflection artifacts in OA images by assuming an equivalent impulse function.^67^ Generally, dual-modality systems based on single-element transducers for endoscopic^68,69^ and sub-surface^70^ imaging have been reported and found wide applications in multi-parametric brain imaging,^21^ melanoma staging,^71^ or skull bone morphogenesis and angiogenesis^64,65^ in mice.

**Fig. 2:**
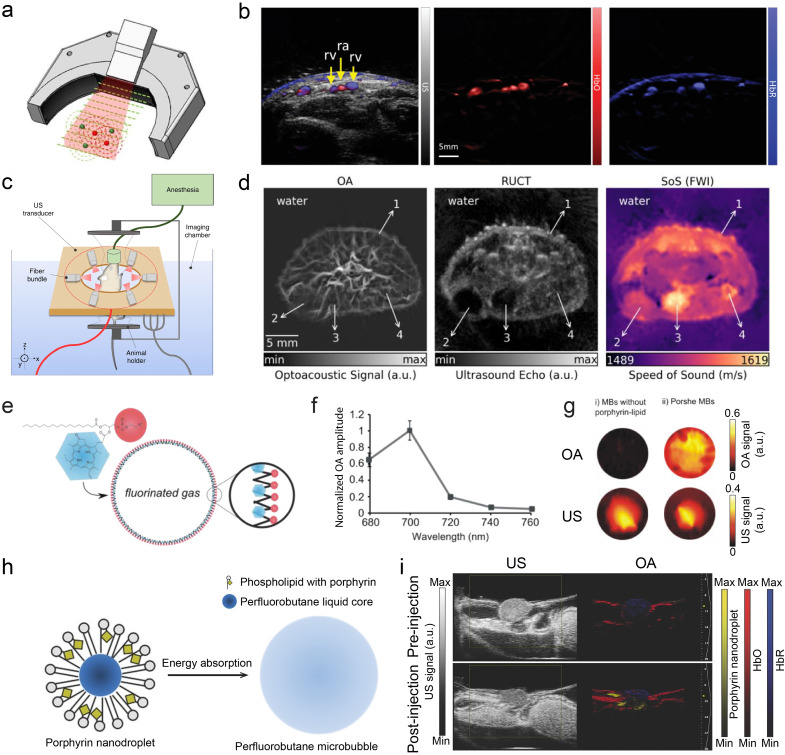
Dual-mode OA and US imaging. (a) Hybrid OA–US imaging probe with a multi-segment array. The linear segment with low inter-element pitch is optimal for B-mode image formation in the pulse-echo US mode whereas lateral concave segments provide broad angular tomographic coverage for accurate OA image rendering. Reprinted with permission from ref. 80. Copyright 2017 AIP Publishing. (b) OA and US images of the human wrist. From left to right: compounded OA–US image along with the maps of HbO and HbR. Reprinted with permission from ref. 83. Copyright 2017 IEEE. (c) Transmission–reflection optoacoustic ultrasound (TROPUS) computed tomography. A full-ring ring array incorporating fiber bundle outputs on both sides was used to hybridize OA tomography with both reflection-mode and transmission-mode US. Reprinted with permission from ref. 23. Copyright 2019 Springer Nature. (d) Representative OA image, reflection-mode US image, transmission-mode SoS image acquired from a cross section of the tumor region in a mouse. Reprinted with permission from ref. 86. Copyright 2020 Elsevier. (e) Schematic of porphyrin shell microbubbles with measured OA spectrum in (f). (g) OA (top) and US (bottom) images of microbubbles without and with porphyrin–lipid in a plastic phantom. Reprinted with permission from ref. 88. Copyright©2012, American Chemical Society. (h) Design of phase-change porphyrin nanodroplets. (i) Image enhancement of both US and OA images of HT1080 tumor in a chicken embryo before and after injection of porphyrin nanodroplets. Reprinted with permission from ref. 56. Copyright 2015 John Wiley and Sons.

Dual-modal OA–US tomographic systems operate instead on a macroscopic imaging scale by employing linear^19,72–75^ or concave transducer arrays^76–79^ in conjunction with broad (unfocused) illumination through a fiber bundle or mirrors.^73^ Dedicated multi-segment array configurations have been proposed to deliver optimal performance in both OA and US modes^22,80–82^ (Fig. 2(a)). Dual-modality imaging with spectrally unmixed HbO and HbR blood components superimposed on pulse-echo US images have extensively been employed in both small animal and clinical investigations (Fig. 2(b)).^83–86^ Several configurations have been reported to combine OA and transmission-mode US imaging, which is additionally capable of rendering speed of sound (SoS) and acoustic attenuation (AA) maps of tissues^23,87^ (Fig. 2(c)). Fully coregistered OA, reflection- and transmission-mode US images of mice were shown to provide highly complementary multi-parametric readings related to disease state and progression in mice, *e.g.* in the context of tumor growth^86^ (Fig. 2(d)) or fatty liver disease.^87^

### OA–US contrast agents

Contrast-enhanced OA–US imaging has been achieved with several types of dual-mode contrast agents. These can be broadly classified into two categories: microbubbles and droplets (Table 1). Microbubbles consisting of a gas–shell structure are routinely employed as US contrast agents. OA contrast has been additionally introduced by incorporating optical-absorbing dyes or nanoparticles (NPs), such as black ink, methylene blue, or porphyrin, onto their shell,^54–56,88^ or by encapsulating nanoparticles (*i.e.*, ink, gold nanorods) within a gas cavity.^89,90^ For instance, engineered microbubbles featured with a porphyrin shell have been proposed (Fig. 2(e)), exhibiting peak optical absorption within the near-infrared (NIR) window (Fig. 2(f)). The enhanced OA signal from porphyrin shell microbubbles, when compared to unmodified microbubbles and microbubbles mixed with free porphyrin, confirms their potential as a dual-modal contrast agent (Fig. 2(g)).^88^ Nanodroplets and microdroplets can also be designed as a core–shell structure, characterized by liquid core formed with perfluorocarbon (PFC) or perfluorobutane (PFB). Different types of photo-absorbers, including indocyanine green (ICG), plasmonic NPs, and porphyrin have been integrated into the core^54,91^ or the shell.^56^ Upon exposure to light radiation or high rarefactional pressure,^56^ nanodroplets can undergo a phase change transforming them into microbubbles, thereby enhancing the US contrast (Fig. 2(h)). The small size of these nanodroplets (<200 nm) allows them to extravasate and accumulate in tumor regions *via* the enhanced permeability and retention (EPR) effect.^92,93^ The dual contrast of the accumulated droplets was exploited for a better characterization of the tumor microenvironment (Fig. 2(i)). Besides simple shell structures, multilayer structures have also been employed, as exemplified by hydrogel microdroplets encapsulating conjugated polymer NPs as photo-absorbers.^59^ The NPs generate OA signals while hydrogel-oil-aqueous layers with varied acoustic resistance provide US contrast. In addition to tumor imaging, dual-modal OA–US contrast agents have been used for urinary bladder,^94^ and pancreas imaging.^91^ Despite promising proof-of-concept studies, many of the reported OA–US contrast agents have not been tested *in vivo*,^54,88–90^ presumably due to biostability or biocompatibility issues. The ongoing development and refinement of these contrast agents remains an active area of research, aiming at achieving stronger OA and US responses, improved targeting functions, extended circulation time, and biodegradability.

**Table 1:** Dual-modal OA–US contrast materials

Modality	Type	Absorber location	Contrast agents	Absorption (nm)	Application	Ref.
OA–US	Microbubbles	OA shell	Methylene blue MBs	675	Urinary bladder (rat)	94
			Black ink MBs	1064		
			Porphyrin shell MBs	700	Phantom imaging	88
		OA core	AuMBs	760	Phantom imaging	89
			Encapsulated-ink PLGA MBs	767	Phantom imaging	90
	Droplets	OA core	ICG-loaded PFC nanodroplet	∼800	Phantom imaging	54
			PAnDs	780	Pancreas (mouse)	95
			Hydrogel-based microdroplets	750	Tumor imaging	59
		OA shell	Porphyrin nanodroplet	705	Tumor imaging (chicken embryo)	56

## Optoacoustic and fluorescence imaging

FL imaging is a mainstay technique in biological discovery owing to its high sensitivity and specificity in detecting targeted molecular labels and activity indicators. The depth covered with FL microscopy systems is typically restricted to <1 mm, mainly owing to the strong photon scattering in living biological tissues. In contrast, OA imaging maintains high resolution imaging performance at millimeter to centimeter scale depths, owing to the insignificant scattering of US waves relative to light. Nevertheless, OA imaging can greatly benefit from the versatile molecular contrast of FL-based techniques. FL imaging assisted with FDA-approved dyes like ICG is routinely used in the clinics, *e.g.* in ophthalmology or surgical guidance applications. Most optical dyes provide excellent OA contrast, making them promising candidates for multimodal OA–FL applications.

### OA–FL imaging systems

**Fig. 3:**
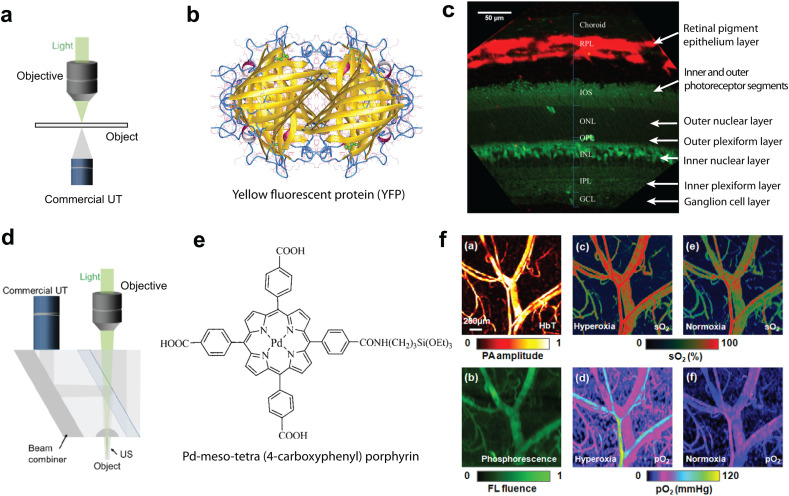
Dual-modal OA–FL microscopy. (a) Typical design employing light and US collection from opposite directions. (b) Structure of yellow fluorescence protein (YFP) which was employed to label dipolar cells in the retina. (c) Sectioned retina slice imaging of transgenic mice after superimposing the YFP labeled bipolar cells and the retinal pigment layer acquired with OA microscopy. Reprinted with permission from ref. 98. Copyright 2014 SPIE. (d) System design for dual-modality signal detection from the same direction. An acoustic-optical beam splitter is inserted between the objective and the imaged object. (e) Pd–*meso*-tetra(4-carboxyphenyl)porphyrin was employed as the probe to image oxygen partial pressure (*p*O_2_). (f) Multiparametric vascular imaging in the mouse ear under hyperoxia and normoxia conditions with a dual-modality OA–CFM system. Top: OA intensity and sO_2_ maps. Bottom: FL intensity and *p*O_2_ maps. Reprinted with permission from ref. 100. Copyright 2011 Optica Society.

Scanning OA and FL microscopy may share a common excitation path based on focused light beams. Hybrid OA–FL imaging systems then allow for a separate collection of the generated US and FL responses. This can been achieved by placing light sensors and US transducers on the opposite sides of the sample (Fig. 3(a)), which enabled multimodal integration of OA with nonlinear optical microscopy methods, such as two-photon microscopy (2PM), second-harmonic generation (SHG) microscopy, and third harmonic generation (THG) microscopy.^96,97^ Mechanical scanning is generally used to acquire two-dimensional OA images, while optical scanning with a galvo mirror is the common approach in nonlinear modalities. To this end, OA microscopy has been combined with confocal fluorescence microscopy (CFM) and 2PM based on a commercial FL microscope, thus enabling sectioned retinal slice imaging in transgenic mice. The FL mode was used to detect yellow fluorescence protein (YFP, Fig. 3(b)) labeled bipolar cells, while melanin in the retinal pigmented layer provided the absorption contrast in OA images^98^ (Fig. 3(c)). A different multimodal OA–FL approach employs an epi-illumination approach with both light and US detectors positioned on the same side to facilitate imaging of thicker specimen. It has been implemented with a customized miniaturized US transducer inserted between the objective and the imaged object.^29^ The compact design employs a high numerical aperture (NA) objective with minimal interference to the US transducer. This hybrid OA–CFM system enabled imaging of 4T1 breast cancer cells labeled with FL proteins in a xenograft tumor model with surrounding vascular network being depicted in the OA mode.^29^ Another type of the hybrid combination involved the use of a mechanically-scanned acoustic-optical beam splitter, which was inserted between the objective and sample to reflect the generated OA waves while allowing the detection of back-scattered FL^99^ (Fig. 3(d)). By injecting oxygen sensitive Pd–*meso*-tetra(4-carboxyphenyl)porphyrin phosphorescent probe (Fig. 3(e)), mapping of both blood hemoglobin sO_2_ and tissue oxygen partial pressure (*p*O_2_) has been demonstrated under normoxia and hyperoxia conditions^100^ (Fig. 3(f)). The sO_2_ map was acquired with dual-wavelength excitation at 570/578 nm (Fig. 3(f), top), which correlated well with the *p*O_2_ values derived from the FL lifetime of the probe measured with CFM (Fig. 3(f), bottom). Transparent US transducer (TUT) technology has opened new possibilities for multimodal OA–FL imaging using on-axis excitation, further enabling quadruple modality US, OA, optical coherence tomography (OCT), and FL fusion imaging.^101^ Optically-transparent micro-ring resonator (MRR)-based US transducers have also been developed,^102^ offering high US detection sensitivity, a wide angular detection angle, and miniature (submillimeter) size. Fabrication of a MRR directly on a microscope coverslip facilitated the hybrid OA–CFM microscopic imaging with a commercial inverted microscope platform, which was subsequently used to image retinal pigment epithelium samples *ex vivo* with cellular resolution.^103^

**Fig. 4:**
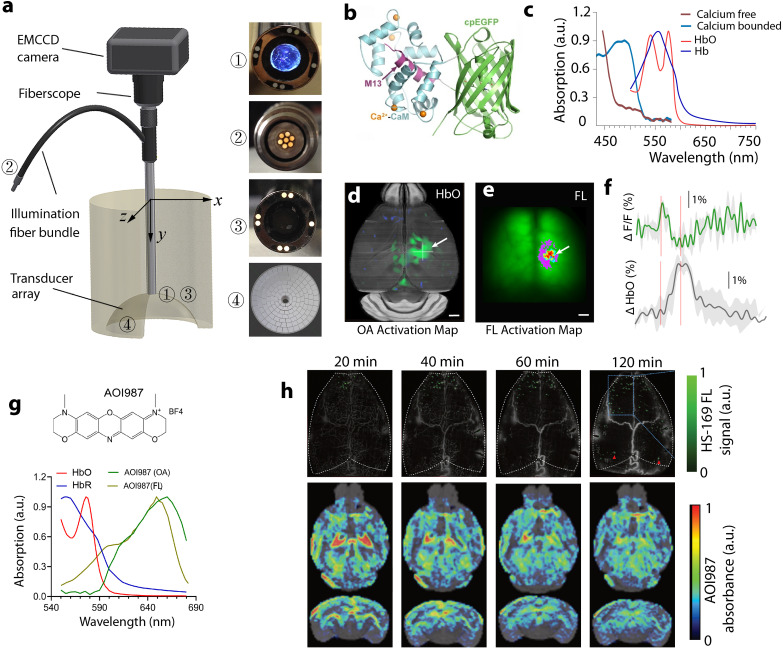
Simultaneous OA and FL imaging for biomedical applications. (a) Schematic of a hybrid OA-epiFL system. Reprinted with permission from ref. 26. Copyright 2017 Optica society. (b) Structure of GCaMP fluorescent protein bound to Ca^2+^. (c) Absorption spectrum of the GCaMP sensor along with the oxy- and deoxy-hemoglobin spectra. (d), (e) OA and FL activation maps from GCaMP-labeled mouse brain in response to electrical hindpaw stimulation. Reprint with permission from ref. 106. Copyright 2022 John Wiley and Sons. (f) The corresponding fractional signal changes in a selected point. (g) Chemical structure and extinction spectrum of an oxazine dye derivative AOI987 probe for amyloid-β targeting as measured by OA and spectrophotometer along with the oxy- and deoxy-hemoglobin spectra. (h) FL signal of the luminescent conjugated oligothiophene HS-169 signal recorded at single plaque resolution in arcAβ mouse cortex at 20, 40, 60, 90, and 120 min after dye administration by the large-scale multi-focal illumination (LMI) fluorescence imaging technique. The corresponding OA signal distribution of the oxazine dye derivative AOI987 across the whole mouse brain, as recorded by volumetric multi-spectral optoacoustic tomography (MSOT), is shown in coronal, and horizontal maximal intensity projection (MIP) views. Reprinted with permission from ref. 107. Copyright 2022 Springer Nature.

Contrary to scanning FL microscopy, widefield epifluorescence (epiFL) imaging is characterized by high temporal resolution and large (centimeter scale) field-of-view (FOV) matching well those achieved with tomographic OA systems. Yet, a combined OA-epiFL imaging is hampered by the fact that optimal tomographic OA image acquisition implies signal collection over a large angle surrounding the object thus limiting the physical space for recording FL responses. Simultaneous OA-epiFL imaging of thin samples in transmission mode was accomplished with a 60-element hemispherical transducer array and a CCD camera arranged on the opposite side of the sample.^25^ More recently, OA-epiFL imaging in reflection mode was achieved using an electron multiplying charge-coupled device (EMCCD)-based fiberscope inserted in a central aperture of a 512-element hemispherical transducer array^104^ (Fig. 4(a)). Excitation of both OA and FL responses was performed with a nanosecond pulsed laser that triggered simultaneous EMCCD and US data acquisition. Liposomal ICG was used to achieve contrast-enhanced images of the mouse brain *in vivo* with both modalities and increase the circulation time of free ICG.^28,104^ Combined epiFL and OA imaging of sensory-evoked brain activity has been demonstrated by simultaneously capturing hemodynamics and calcium activity using genetically-encoded calcium indicator GCaMP6f^105^ (Fig. 4(b) and (c)). OA-epiFL imaging enabled concurrent measurements of neuronal activity and accompanying hemodynamic responses in mice, thus attaining multiparametric noninvasive characterization of brain activity and neurovascular coupling^106^ (Fig. 4(d)–(f)). When combined with Aβ-targeted probes, such as the luminescent conjugated oligothiophene HS-169 or oxazine dye derivative AOI987, OA-epiFL imaging facilitates transcranial Aβ detection at multiple scales, all the way from single-plaque resolution in the cortex to whole brain mapping of the plaque load in deep areas such as hippocampus and thalamus^107^ (Fig. 4(g) and (h)). Enhancement in tissue penetration and imaging contrast can be achieved for both OA and FL modalities when leveraging the diminished scattering and autofluorescence in the second near-infrared (NIR-II, 1000–1700 nm) range, as has been shown for non-invasive angiography,^108^ inflammation detection,^109^ tumor diagnosis,^108,110^ and photothermal therapy^111–113^ applications.

### OA–FL contrast agents

OA provides label-free contrast based on optical absorption of endogenous substances, such as oxygenated and deoxygenated hemoglobin, melanin, bilirubin, lipids, and water. OA imaging can also visualize FL substances that thermalize part of the absorbed energy.^27^ Small FL molecules, such as ICG^114^ or other cyanine dyes^115,116^ having absorption peaks in the NIR spectrum, are ideally suited for synergistically exploiting the advantages of dual-modality OA–FL imaging. The enhanced penetration of light in the NIR range enables tracking specifically-labeled biomolecules in small animals at the whole-body level.^117^ Both FL and OA signal intensities are proportional to the extinction coefficient (*ε*) of the contrast material. However, FL intensity scales with the quantum yield (QY) while OA intensity is proportional to 1-QY.^27^ Organic FL dyes with peak absorption in the NIR spectrum commonly exhibit a relatively high *ε* yet low QY, making them particularly suitable for hybrid OA–FL imaging.^60^ Hybrid OA–FL can also be performed with particulate agents, *e.g.* quantum dots (QDs),^118^ gold nanorods (GNRs),^119^ carbon nanotubes,^120^ FL silica NPs,^121^ or liposomes.^122^ In addition, the use of particles composed of organometallic materials,^123^ metal-dye composites,^124^ or metal–polymer composites^125^ have also been explored. A list of common OA–FL dual-modal contrast materials appears in Table 2.

**Table 2:** Dual-modal OA–FL contrast materials

Modality	Category	Subcategory	Contrast agents	Absorption/emission (nm)	Application	Ref.
OA–FL	Organic materials	Small-molecule dyes	PSMAP/ICG NBs	780/820	Prostate cancer	114
			Cy7-1-maltotriose	750/780	Bacterial infection	115
			SAPTN	1024/1128	mildPTT, cancer immunotherapy	126
			MC-PSE	900, 980/940	Glutathione detection	127
			DTP-DPTQ NPs	852/1120	PTT of breast cancer model	111
			CySO_3_-GGT	675/750	Hepatocellular carcinoma	128
			LET-12	1400/1520	Glioblastoma PTT	112
			CyA	602/719	PDT, systemic immunotherapy	129
			IR780-SPhF	780/810	Triple negative breast cancer theranostics	116
			FMP&N-FMP	680/689	PDT, cancer Immunotherapy	130
			P-CyPt	700, 750/710	Cancer theranostics	131
			CTSK-APPA	615, 690/720	Early osteolytic metastasis	132
			AOI987	650/720	Aβ imaging	107
		AIEgens	MPNPs	500–800/650–1100	Cancer immunotherapy, type I/II PDT	133
			AIE-4PEG550 NPs	645/900–1700	Kidney fibrosis	134
			C-NTBD NPs	732/1042	Resection of neuroendocrine neoplasms and SLN	135
		Polymeric nanoprobes	EMT-NPs	>700/643–765	PDX cancer treatment	136
			SPCy	607, 670/720, 800	Neutrophil elastase	137
			P2NPs	920/1120	osteosarcoma PTT	138
			CF-SPNs	580, 775/680, 820	Drug-induced hepatotoxicity monitoring	139
			mPPy@COF-Por	808/501	PDT, PTT of colorectal carcinoma	131
	Inorganic materials	Rare-earth doped NPs	Gd_0.8_Nd_1.2_O_2_S@PVP	680–970/1000–1700	Phantom study	126
		Noble metals	AuPd-BSA CN	400–800/1100–1500	Dual-PDT synergized enzyme catalytic therapy	140
		Quantum dots	PCD	400–900/450–650	Breast cancer model	141
			V_2_C-TAT@Ex-RGD	1000–1350/422	Nucleus-targeted PTT	142
			a-Ag_2−*x*_Cu_*x*_S QDs	635/∼820	PTT of murine hepatoma tumor	143
		Other	AuNNPs–Ag_2_S Ve	400–1200/1250	RT of breast cancer model	110
			mdGC	510, 798/480	PTT of breast cancer model	144
			HSC-2	400–1000/957	PTT/catalytic synergistic therapy	113
	Hybrid materials	Organometallic materials	PhAg NPs	680, 850/675, 730	Chemo-PTT melanoma therapy	123
		Metal-dye composites	CFNPs	760/780–840	Photothermal primed CDT	124
			Cy5-conjugated PEG@AgIONPs	680–924/640	PTT, Thrombosis	145
		Metal–polymeric NPs	Au^0^–Por@FeCO nanosheets	200–1000/630–800	PTT-gas therapy of breast cancer model	125
		Other	AHZ NPs	744/1000–1700	PTT in deep tumors	146
			MTCNs	1048/1080, 1550	H_2_O_2_ imaging, lymphatic metastasis	147
			CRUN	663/545, 655	Tumor microenvironment	148
			HCy5/Cy7-UCNs	640, 780/660–800 (tunable)	Multiple ROS/RNS species sensing	149

**Fig. 5:**
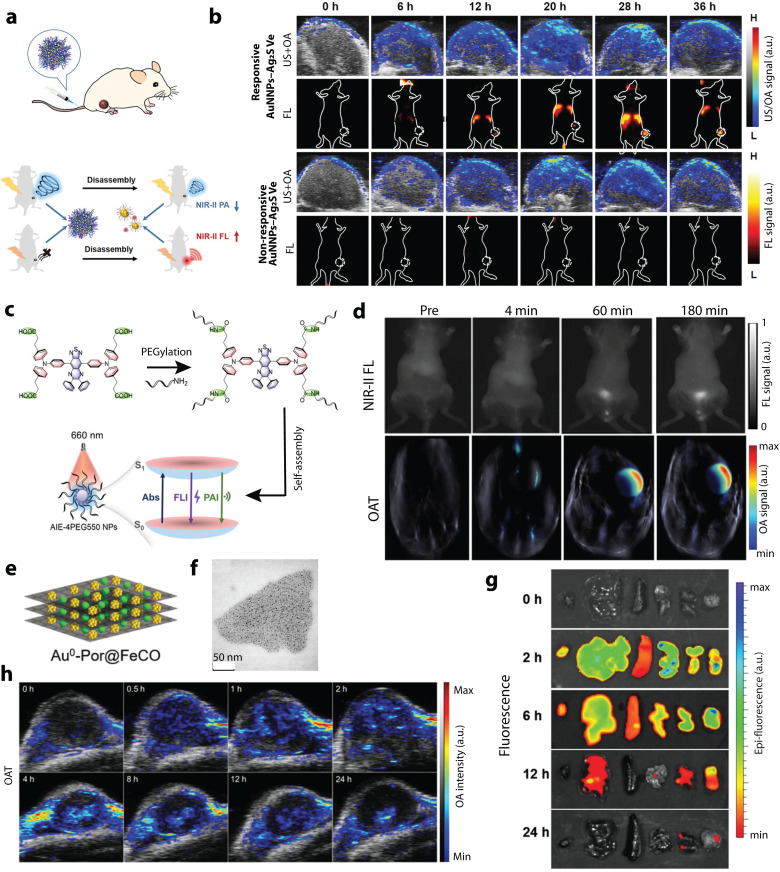
OA–FL dual-modal contrast materials and corresponding applications. (a) Schematic illustration of pH-responsive AuNNPs–Ag_2_S Ve used for activatable FL and OA imaging in the NIR-II window. (b) OA images and FL images of MCF-7 tumor bearing mice after treated with pH-responsive AuNNPs–Ag_2_S Ve and nonresponsive AuNNPs–Ag_2_S Ve through tail vein injection. Reprinted with permission from ref. 110. Copyright 2022 John Wiley and Sons. (c) Molecular design philosophy of self-assembly ultrasmall AIE-4PEG550 NPs and the reconciled photophysical processes. (d) Representative FL and OA images of the bladder of living mice at preinjection, 4, 60 and 180 min after injection of AIE-4PEG550 NPs. Reprinted with permission from ref. 150. Copyright 2022 John Wiley and Sons. (e) and (f) Structure and bright-field TEM image of Au^0^–Por nanosheets. (g) *Ex vivo* fluorescence imaging of 4T1 tumor-bearing mice after intravenous injection of Au^0^–Por. (h) Time-lapse OA images of the 4T1 tumors after intravenous injection of Au^0^–Por suspension. Reprinted with permission from ref. 125. Copyright 2023 John Wiley and Sons.

To this end, the high versatility of OA–FL imaging has been exploited in a number of biomedical applications. A small molecule termed MC-PSE, which is synthesized by utilizing phenylselenophenols to replace the median chlorine atom of an anionic cyanine dye, could self-assemble into J-aggregates in an aqueous solution and disassemble when activated by glutathione. This triggers OA–FL signal fluctuations reflecting the presence of glutathione in cancer.^127^ A synthesized IR780 derivative (IR780-SPhF) also induces ferroptosis-mediated cell death after accumulating in tumors with a highly elevated glutathione level.^116^ The reduced light scattering in NIR-II facilitates imaging with deep-tissue. However, design and synthesis of organic probes emitting light in this range remains challenging. NIR-II fluorophores DTP-DPTQ and LET-12 exhibited good performance in imaging-guided photothermal therapy of breast and glioblastoma tumors.^111,112^ Normally, OA–FL dyes have balanced absorption and emission. However, aggregation-induced emission luminogens (MTPE-TT, AIE-4COOH and C-NTBD) feature boosted FL and OA intensity simultaneously, benefiting multifunctional image-guided self-synergistic immunotherapy,^133^ early diagnosis of renal dysfunction (Fig. 5(a) and (b)),^134^ accurate detection of neuroendocrine neoplasms, and intraoperative sentinel lymph nodes (SLNs) dissection.^135^ Contrary to small molecules, semiconducting polymer particles offer structural flexibility for efficient design of activatable OA–FL probes. For example, semiconducting polymer nanoprobe SPCy initiates its OA and ratiometric FL imaging after reacting with tumor-associated neutrophils.^137^ In addition, the first reported covalent organic framework (COF)-based biomimetic nanomotor composed of a polypyrrole (PPy) core and a porphyrin–COF shell provided a new design strategy of a single multifunctional platform for imaging-guided combined photothermal therapy (PTT) and photodynamic therapy (PDT).^131^

Compared with organic contrast agents, the multimodal OA–FL inorganic materials, including Rare-earth (RE) particles, carbon dots, QDs, and noble nanocomposites, are of interest due to their good photostability, broad absorption band, and long FL lifetime. Rare-earth oxysulfide (Gd_2_O_2_S) NPs have shown excellent optical performance and low cytotoxicity.^126^ Vanadium carbide (V_2_C) QDs modified with TAT peptides and packaged into engineered exosomes could realize nucleus targeting and further destroy tumor cells by low-temperature photothermal therapy.^142^ Ultrasmall carbon dots without excess modification could further penetrate deeper into tumors for improved therapeutic outcomes.^141^ As a novel multifunctional agent, AuPd-BSA CN benefited from its strong NIR absorption and FL intensity in the NIR-II spectrum to guide type I/type II PDT performance. Furthermore, different types of inorganic materials have been integrated together for dual-modal OA–FL imaging in the NIR-II window, such as zeolite–carbon-based nanozymes and nanogapped gold nanoparticles (AuNNPs)–silver sulfide (Ag_2_S) vesicles. These enabled prolonged blood circulation times and enhanced tumor accumulation, thus providing enhanced diagnostic information with high resolution (Fig. 5(c) and (d)).^110,113^

Other sophisticated strategies have been explored to develop advanced OA–FL contrast agents. The FL molecule Pheophorbide-a was loaded into silver NPs that could be activated by NIR light to mediate reactive oxygen species (ROS)-dependent apoptotic cell death to treat melanomas.^123^ According to a simple reaction between tetrachloroaurate and *meso*-tetra(pyridyl)porphyrin hydrochloric solution, Au^0^–Por nanosheets served as the carrier of carbon monoxide-releasing molecules for image-guided gas therapy. The time-to-peak delay of the OA/FL signals between the kidneys and tumor indicates that the nanosheets are accumulated in the tumor by passive targeting, while the quick degradation and excretion by digestive organs corroborates their biodegradability and improved biosafety (Fig. 5(e)–(h)).^125^ Liposomes, approved as a drug delivery system for clinical use, have been widely used to load drugs, dyes, DNA, and other molecules for multifunctional applications. Through loading of Fe^2+^, 2,2′-azino-bis(3-ethylbenzothiazoline-6-sulfonic acid)diammonium salt, citric acid, and polyethylene glycol (PEG)-modified lanthanide downconversion nanophosphors in the aqueous core and encapsulating IR1048 in the lipid bilayer, liposome-based microenvironment-tailored catalytic nanoprobes were allowed to do accurate and sensitive detection of H_2_O_2_*in vivo via* bimodal OA–FL ratiometric imaging in the NIR-II window.^147^ Different from conventional contrast materials employing “on or off” concept, the lanthanide-doped upconversion nanocrystals (UCNs) have been extensively applied in biosensing, molecular imaging, and nanomedicine. Owing to their extraordinary capability to convert NIR photonic excitations into multiplexed emissions ranging from ultraviolet to NIR windows, UCNs can ideally realize a precise interrelation and meet complex biological demands by fitting different sensing moieties into one rationally integrated nanomatrix to simultaneously read out numerous analytes, *e.g.* ROS and reactive nitrogen species (RNS), in highly complex and dynamic living environments.^139,148,149,151^ By taking advantages of multiplexing luminescence of UCNs and two specific ROS- and RNS-sensitive NIR cyanine fluorophores, simultaneous screening of various redox species and dynamic profiling of their intricate correlations with pathophysiological implications was achieved.^149^

## Optoacoustic microscopy and optical coherence tomography

OA microscopy and OCT share a number of common and complementary features in terms of contrast, penetration depth, and spatial resolution, so that more comprehensive tissue characterization is achieved by combining these two modalities. By simultaneously rendering depth-dependent optical scattering profiles, volumetric structural information, chromophore bio-distribution maps, flow velocity values, polarization properties, and temperature maps, the combination between OA and OCT can impact a broad range of applications in oncology, neurology, dermatology, and ophthalmology, both in preclinical and clinical settings.^35,57,152–155^

### OA–OCT imaging systems

**Fig. 6:**
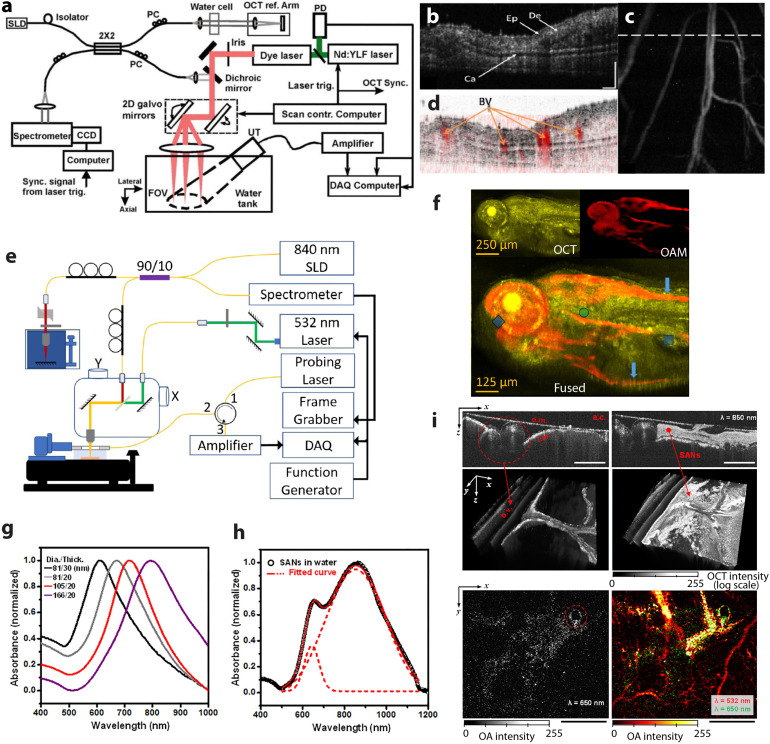
Hybrid OA–OCT imaging. (a) Schematic of the integrated OCT and OA microscopy system in reflection mode. (b) OCT B-scan of the mouse ear *in vivo*. (c) The corresponding top-view OA image with the dashed line indicating location of the OCT B-scan slice; (d) fused OCT and OA B-scan images at the same location. Ep, epidermis; De, dermis; Ca, cartilaginous backbone; BV, blood vessels; bar, 200 μm. Reprinted with permission from ref. 34. Copyright 2009 Optica society. (e) Schematic of the reflection mode OA–OCT system based on a rigid akinetic Fabry–Perot etalon; (f) images of a 120 h post-fertilization (hpf) zebrafish larva. Reprinted with permission from ref. 157. Copyright 2017 Optica society. (g) Measured extinction curves of the aqueous solutions of the Au nanodisks. (h) Measurements for the extinction curves of the aqueous solutions of the SANs. (i) *In vivo* application of the SANs for bimodal imaging of chicken embryos. The top two images show OCT images of a 5 day-old developed chick embryo (left) before and (right) after injection of SANs. Bottom row: OA images of (left) the injected SANs only and (right) the injected-SANs overlapped with blood flows. The SANs and vasculature images were observed by illuminating at 650 and 532 nm wavelengths, respectively. Reprinted with permission from ref. 158. Copyright 2017 American Chemical Society.

Much like for OA–FL imaging, early implementation of hybrid OA and spectral domain OCT was achieved in transmission mode with light delivery and US detection performed from the opposite sides of a thin sample. Light was coupled into the platform through a single-mode optical fiber and focused into the object with a microscope objective.^152^ In contrast, reflection mode (epi-illumination) OA–OCT systems share the same optical scanning method for both OA and OCT, with an US detector positioned at the same side to enable an intrinsically-registered imaging performance, as demonstrated *e.g.* by simultaneous visualization of the microanatomy and microvasculature of the mouse ear *in vivo* (Fig. 6(a)–(d)).^34^ The opacity of standard US transducers poses challenges for the hybridization between OCT and OA, which was typically achieved by positioning an unfocused US transducer obliquely with respect to the optical axis (Fig. 6(a)), thus causing loss of sensitivity for OA signal detection. A modified design of an akinetic sensor with a large translucent imaging window and a thickness of only 1 mm was reported for dual-modal OA–OCT. The *in vivo* images of zebrafish larvae produced with this system demonstrate its potential in biomedical research (Fig. 6(e) and (f)). Alternatively, TUTs represents a promising avenue for hybridizing OA imaging with other optical imaging methods by enabling on-axis excitation and detection, as previously described in the hybrid OA–FL section. In the same work, seamless integration between US, OA, OCT, and FL imaging was reported based on co-axial illumination through a TUT, further incorporating a spherical acoustic focusing lens combined with multiple light sources, namely, two pulsed lasers for spectroscopic OA imaging, a continuous wave (CW) laser for FL imaging, and a superluminescent light-emitting diode for OCT.^101^ This quadruple fusion imaging system was shown to provide multiparametric visualization of ophthalmic injuries and neoplastic lesions in rodents. OA imaging methods are well-established for quantifying blood oxygen saturation, which can be complemented with flow measurements provided by Doppler OCT. This provides a powerful means for characterizing blood flow in cardiovascular diseases, such as stroke, hemorrhages, vascular occlusions, as well as other pathologies with flow stasis such as tumors.^35,156^ It is also expected to open a broad range of applications in studying angiogenesis, tissue inflammatory, or healing responses.

### OA–OCT imaging contrast agents

The sensitivity and specificity of both OA and OCT can be improved by adding exogenous contrast agents, thus extending the application scope of both modalities from imaging the intrinsic tissue contrast to specific molecular and cellular observations. Among different types of contrast materials, gold nanoparticles (AuNPs) generate signals detectable with both modalities.^159,160^ Owing to the surface plasmon resonance, AuNPs have unique optical properties including strong optical absorption and scattering, making them excellent candidates for dual-mode OA–OCT imaging. AuNPs also have excellent optical tunability as well as bio-, photo-, and thermal-stability. Physically synthesized gold nanodisks (SANs) have been proposed as dual-modal OA–OCT contrast agents due to the excellent tunability of their optical properties, including resonant wavelengths, absorption-to-scattering ratio, and responsiveness to random incident light. By changing the nanodisk's thickness, it is possible to shift its resonant frequency while maintaining the lateral size and achieve excitation within a broad spectral window (Fig. 6(g)). Furthermore, different-sized disks inside the SAN can interact with different wavelengths of the incident light, as evidenced by the extinction spectrum measured from the SAN aqueous solution and fitted by the envelope curve of two Gaussian peaks (Fig. 6(h)). In a chick embryo study SANs could be spectrally differentiated from other tissue chromophores by OA, whilst OCT enabled correlating the NPs distribution profiles with the surrounding tissue structures (Fig. 6(i)).^158^ Gold nanoshells are other type of AuNPs have also been used to enhance the OCT contrast in cells and improve microangiographic images. In this way, the signal from the retinal and choroidal vessels in living rabbits was enhanced by up to 82% and 45% for OA and OCT, respectively.^57^ Importantly, conventional OCT contrast generating moieties such as hemoglobin, gold nanorods and plasmonic NPs,^161–166^ liquid-filled microspheres^167^ and absorption-based NIR dyes^168–170^ can also serve as OA contrast agents if made to exhibit suitable light absorption properties. Recently introduced gas vesicles (GVs), a class of naturally evolved gas-filled protein nanostructures, can serve as genetically encodable OCT contrast agents. GVs have yet to be used for OA imaging, but might be a promising candidate for dual-modal OA–OCT applications as the light absorption of the protein is substantially different from the surrounding aqueous environment.^171^ A summary of dual-modal OA–OCT contrast agents is provided in Table 3.

**Table 3:** Dual-modal OA–OCT contrast materials

Modality	Category	Subcategory	Contrast agents	Peak absorption (nm)	Application	Ref.
OA–OCT	Inorganic materials	Gold particles	Stacked gold nanodisks (SANs)	670–830	Chick embryo imaging	158
			PEG-AuNPs	520	Retinal and choroidal blood vessels imaging	57
			Gold nanorod	700	Choroidal neovascularization imaging	155
			Gold nanostars	650	Choroidal neovascularization imaging	154
		Gold clusters	CGNPs (clusters)	650	Choroidal neovascularization imaging	172

## Optoacoustic and Raman scattering microscopy

Raman scattering microscopy (RSM) is a well-established technique for detecting both endogenous and exogenous markers with high specificity, based on vibrational and rotational transitions of molecular structures. The relatively weak intrinsic Raman scattering signal can be amplified with the surface-enhanced Raman scattering (SERS) and the surface-enhanced resonance Raman scattering (SERRS) effects. RSM offers complementary molecular contrast when paired with OA imaging. However, concurrent OA-Raman measurements are impeded by several factors. Firstly, due to the small cross-section of Raman scattering, RSM suffers from inherently weak signals, necessitating lengthy integration times for each measurement point whereas OA microscopy recordings over large FOVs are typically done within seconds or minutes. Secondly, in coherent RSM two ultrafast pulsed lasers in femtosecond or picosecond range are used while OA usually employs a different type of a nanosecond pulsed laser to effectively induce the OA effect. Finally, RSM and OA have different contrast mechanisms and do not share common information that can facilitate image co-registration. Given the differences in their temporal and spatial resolutions, signal excitation/detection schemes, and contrast mechanisms, OA-Raman imaging has been performed by sequential scanning with both modalities. Most of the recent work in this topic has focused on the development of dual-modal OA-Raman contrast agents, particularly for tumor diagnosis purposes to enhance clinical management of cancer.

### OA-Raman contrast agents

**Fig. 7:**
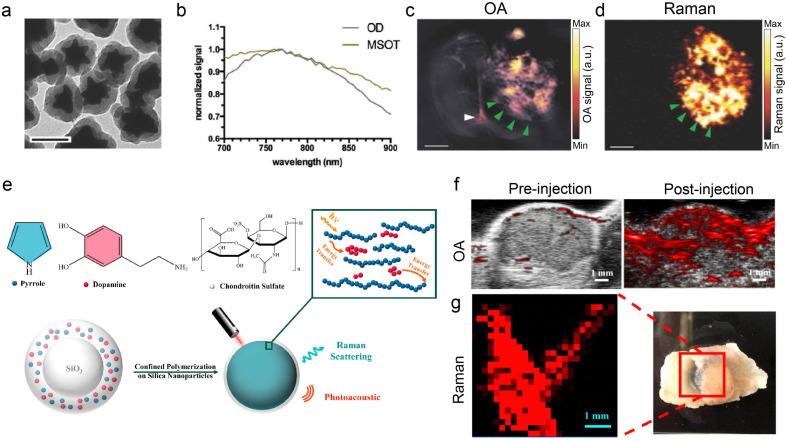
OA-Raman dual-modal contrast materials and corresponding applications. (a) Transmission electron microscopy image of SERRS-MSOT-nanostars. (b) The corresponding optical absorption spectrum of the nanostars. (c) and (d) MSOT and SERRS imaging of the nanostars in a glioblastoma (GBM) tissue section. Reprinted with permission from ref. 33. Copyright 2018 John Wiley and Sons. (e) Schematic of SiO_2_-CS@PPy-PDA nanoparticle and its energy transfer model. (f) OA image of the tumor region before and after the intratumoral injection of SiO_2_-CS@PPy-PDA in an A549-tumor-bearing mouse under 700 nm illumination. (g) Raman image of a resected tumor tissue, acquired with 785 nm excitation. Reprinted with permission from ref. 32. Copyright 2018 American Chemical Society.

To harness the advantages of excellent molecular specificity of Raman imaging and deep penetration of OA imaging, several dual-modal OA-Raman contrast agents have been introduced, primarily in the form of core–shell composites (Table 4). Nanostars for combined SERRS and multi-spectral optoacoustic tomography (MSOT) imaging were produced by encapsulating light-absorbing IR780 dye onto a gold nanostar core (Fig. 7(a)), which resulted in a light absorption peak at ∼770 nm (Fig. 7(b)). The proposed SERRS-MSOT-nanostars were subsequently employed for dual-mode imaging of glioblastoma tissue sections (Fig. 7(c) and (d)).^33^ GNRs are another type of nanomaterial providing high SERS signal and a tunable optical absorption cross section. When functionalized with SERS reporters such as IR792, the modified GNRs manifest a different Raman spectrum, and the absorption peak could be tuned by changing the aspect ratio and particle size for optimized OA performance.^30^ Besides, a ratiometric dual-modal contrast agent, termed AuNNR@MSi-AuNPs, was designed based on a core–satellite nanostructure by incorporating additional standard OA-Raman reporters located on mesoporous silica-coated nanogapped gold nanorods (AuNNRs) for optimal OA and Raman imaging. Specifically, the ratiometric OA-Raman readings between standard reporters (OA: gold nanorod; Raman: 2-naphthalenethiol) and reference reporters (OA: 2,2′-azino-bis 3-ethylbenzothiazoline-6-sulfonic acid; Raman: 4-mercaptobenzoboric acid) facilitate quantitative detection of H_2_O_2_ in subcutaneous tumor models in mice and knee osteoarthritis in rabbits.^173^ Another approach involves doping polydopamine (PDA) with the semi-conducting polymer PPy on the supportive SiO_2_ templates, shown in Fig. 7(e). PDA offers high absorption and tunes the optical bandgap energy for enhanced Raman signals and diminished FL background due to the resonance Raman effect. Both macromolecules are deposited on a SiO_2_ template to form a SiO_2_-CS@PPy-PDA nanoparticle, using chondroitin sulfate as the stabilizer. The enhanced OA and Raman signals were validated in images of tumor regions in A549-tumor-bearing mice (Fig. 7(f) and (g)).

**Table 4:** Hybrid OA-Raman contrast materials

Modality	Category	Subcategory	Contrast agents	Absorption (nm)	Application	Ref.
OA-Raman	Organic materials	Polymeric nanoprobes	SiO_2_-CS@PPy-PDA	650	Tumor imaging	32
	Inorganic materials	Nobel metals	AuNNR@MSi-AuNPs	750	Tumor, knee osteoarthritis	173
	Hybrid materials	Metal-dye complexes	SERRS-MSOT-nanostars	770	Tumor imging (glioblastoma)	33
		Other	GNR with SERS reporter	661/698/756	Tumor imaging (ovarian cancer)	30

## Combining optoacoustic and magnetic resonance imaging

Magnetic resonance (MR) imaging is a well-established anatomical and functional imaging tool featured with excellent soft tissue contrast and whole-body coverage, both in small animals and humans. As a non-invasive and non-ionizing modality, it is widely used in preclinical and clinical applications and is often the method of choice in oncological and brain investigations.^174^ Like any other imaging modality, MRI is afflicted with a number of shortcomings, including high procurement and maintenance costs, slow imaging speed for high-resolution interrogations, and limited molecular sensitivity. Functional MR imaging (fMRI) based on the blood-oxygen-level-dependent (BOLD) signal has become the gold standard to study brain function. Likewise, fMRI suffers from limited ability to capture fast neuronal responses owning to the indirect hemodynamic readings provided by the BOLD signal.^175^ Advanced molecular MRI approaches have been proposed, yet failing to provide high spatio-temporal resolution readings with adequate sensitivity.^176^ In contrast, OA offers fast imaging performance with higher molecular sensitivity but limited penetration depth and relatively poor soft tissue contrast. Powerful complementary advantages could therefore be unlocked with dual-modal OA–MR imaging. The excellent soft tissue contrast provided by MR may serve as anatomical reference for OA, while the multiparametric hemodynamic readings retrieved with OA can complement and cross-validate the BOLD signal, which is currently assumed to be primarily sensitive to deoxygenated hemoglobin. Generally, this multimodal approach can impact many biomedical research fields and improve the diagnostic and therapeutic monitoring capabilities of the standalone modalities *e.g.* in breast cancer or cardiovascular disease imaging applications. Considering that OA has recently been shown capable of imaging the human brain,^177,178^ the multimodal OA–MR combination may potentially have an impact on clinical neuroscience.

### OA–MR imaging systems

**Fig. 8:**
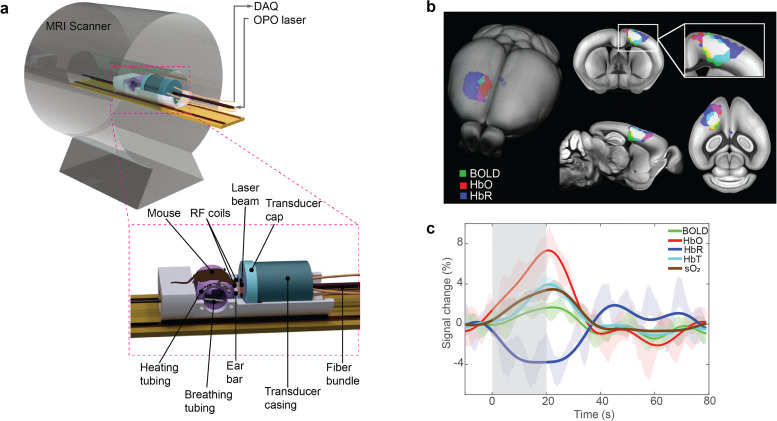
Label-free functional OA–MR imaging. (a) Layout of a hybrid magnetic resonance optoacoustic tomography (MROT) scanner enabling concurrent MR and OA readouts. Reprinted with permission from ref. 183. Copyright 2023 Elsevier. (b) Averaged fractional signal intensity changes of BOLD and multi-parametric OA hemodynamic components in the mouse brain following peripheral sensory stimulation. (c) Superposition of the BOLD, HbO, and HbR activation maps onto the mouse brain atlas. The white color indicates the overlapping region of the three components. Reprinted with permission from ref. 45. Copyright 2022 John Wiley and Sons.

The high magnetic field strength used by MR scanners combined with the strong field gradients generated by radio-frequency (RF) pulses hampers efficient implementation of the dual-modality OA and MR imaging, which has mainly been achieved *via* stand-alone acquisitions.^40,44,179–181^ Accurate image coregistration is crucial for accurately combining, correlating, and validating the information acquired from independent measurements. This has mostly been performed *via* landmark-based methods from sequentially acquired images.^42,182^ However, coregistration is significantly challenged by differences in orientation and tissue deformations resulting from independent acquisitions. Recently, a fully hybrid magnetic resonance optoacoustic tomography (MROT) scanner has been developed featuring an MR-compatible OA tomography module inserted into a 9.4 T preclinical MR scanner bore (Fig. 8(a)).^43^ MROT provides concurrent dual-modal *in vivo* anatomical and functional acquisitions, thus averting the complexity of the coregistration problem with simultaneously acquired images.^183^ The mutual interference challenges between the OA and MR acquisitions were mitigated *via* automatic detection of corrupted frames and by using deuterium oxide (heavy water) to couple US waves. Induced hemodynamic responses to an oxygen challenge paradigm were successfully captured with both modalities across the entire mouse brain.^43^ Additionally, highly-correlated changes in BOLD and OA hemodynamic components (HbO, HbR, HbT and sO_2_) (Fig. 8(b)) were observed under an electrical stimulation paradigm.^45^ Stronger percentile changes were measured in the HbO signal (Fig. 8(c)), while BOLD and HbR signals were found to be less sensitive to sensory responses.^45^

### OA–MR contrast agents

**Fig. 9:**
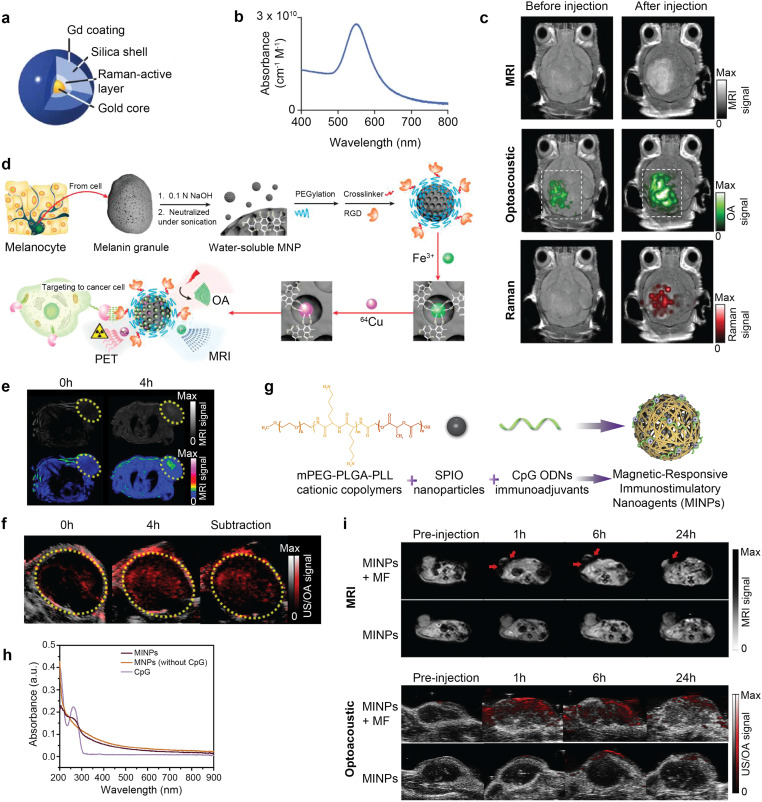
Contrast enhanced OA–MR dual-modal imaging. (a) Structure of a triple-modality OA-MP-Raman NP. (b) Its corresponding optical absorption spectrum. (c) Triple-modality *in vivo* OA–MR-Raman imaging before and after injection of the NP. Reprinted with permission from ref. 31. Copyright 2012 Springer Nature. (d) Synthesis of ^64^Cu-Fe-RGD-PEG-MNP nanoplatform for triple modality OA–MR-PET imaging. (e) OA imaging of mice bearing U87MG tumors *in vivo* pre- and postinjection of ^64^Cu-Fe-RGD-PEG-MNP. (f) *T*_1_-weighted MRI and pseudo-colored MRI *in vivo* pre- and postinjection of ^64^Cu-Fe-RGD-PEG-MNP. Reprinted from with permission ref. 196. Copyright 2014 American Chemical Society. (g) Synthesis of MINPs (CpG@PLGA-PLL-mPEG/SPIO). (h) Optical absorption spectra of the free CpG ODNs, MNPs (without CpG ODNs) and MINPs. (i) Dual-modal OA and *T*_2_-weighted MR images at different time points following injection of MINP with and without application of an external magnetic field. Reprinted with permission from ref. 41. Copyright 2019 Elsevier.

A variety of probes have been developed to enhance performance of the dual-modal OA–MR imaging (Table 5). Of particular interest are nanomaterials providing high optical absorption and altered *T*_1_ or *T*_2_ relaxation times, such as Gd^3+^ chelates, superparamagnetic iron oxide (SPIO) NPs, manganese(ii) chelates, and reporter genes.^184^ Triple-modality MR–OA-Raman imaging was achieved with multimodal NPs composed of a gold core covered with Raman active layer and coated with Gd^3+^ (Fig. 9(a)).^31^ These provided a high longitudinal (*T*_1_) relaxation time (3.0 × 10^6^ mM^−1^ s^−1^), strong optical absorbance (2.75 × 10^10^ cm^−1^ M^−1^) peaked at 540 nm (Fig. 9(b)), and a unique Raman signature. Consecutive MR, OA and Raman imaging revealed EPR-based co-localized accumulation of the NPs in murine glioblastomas following intravenous injection (Fig. 9(c)). Whole-brain localization of the tumor was achieved by MRI, with OA offering high 3D spatial resolution and Raman providing high-sensitivity surface imaging of the tumor margins.

**Table 5:** Dual-modal OA–MR contrast materials

Modality	Category	Subcategory	Contrast agents	Peak absorption (nm)	Application	Ref.
OA-MR	Organic materials	Small molecule	Gd-IR780	790	Tumor apoptosis	185
	Inorganic materials	Metal sulfide	Cu_2−*x*_S NPs	∼1160	Cervical cancer	186
			Cu_2_MnS_2_ NPs	800–1300	PTT of murine sarcoma model	187
		Multimetallic particles	Au-IO NP	530–550	Cell imaging	188
			SPIO@Au	810	Glioblastoma tumors	40
			Nanowontons	700	Mouse imaging	189
			MSIOs	NIR	PTT of hepatocellular carcinoma	190
		Iron particles	MINPs	NIR	Immunotherapy, PTT of breast cancer model	41
			Fe^3+^-PEG-MNP	680–980	MSC labeling	191
		Other	Cobalt at carbon NPs	400–1000	Glioma tumors	192
			Gd–Fe/HCSs	400–1100	Liver imaging	193
			HA-MnO@MSN NPs	N.A.	Tumor oxygen modulation	181
	Hybrid materials	Metal–polymer composites	CP-IO	750	Breast tumor imaging	39
		Other	DDNPs	790	Thrombus diagnosis	194
			PB:Mn nanocubes	808	PTT of breast cancer model	195

Melanin, an endogenous biomarker for melanoma detection providing strong OA contrast, has also been applied in the dual-modal OA–MR setting.^196^ PEGylated melanin NPs (MNPs) were further conjugated with RGD for tumor targeting and chelated to ^64^Cu^2+^ (PET radiolabel) and Fe^3+^ (*T*_1_ contrast agent) ions to form a ^64^Cu–Fe-RGD-PEG-MNP nanoplatform for triple-modality OA–MR–PET imaging (Fig. 9(d)). Hyperintensities in the *T*_1_-weighted (Fig. 9(e)) and OA images (Fig. 9(f)) were produced at the tumor site. Both MR and OA signals were found to be linear with NP concentration, with OA being superior to MR in terms of detection sensitivity. A myriad of other probes have been developed for dual-mode OA–MR diagnostic imaging, including cobalt NPs,^189^ copper sulfide NPs,^186^ copper manganese sulfide nanoplates,^187^ conjugated polymer iron oxide (CP-IO) NPs,^39^ SPIO@Au-labeled mesenchymal stem cells (MSCs),^40^ manganese monoxide nanocomposites (MnO@Au NCs),^197^ PEGylated melanin and iron ions NPs (Fe^3+^-PEG-MNPs),^191^ manganese dioxide coated NPs,^180^ cobalt core/carbon shell NPs,^192^ carbon nanospheres,^193^ caspase-3 activatable Gd-chelated fluorophores,^185^ MnO-hyaluronic acid NPs,^181^ gold/iron oxide (Au–IO) multimetallic NPs^188^ and fluorophore–protein complex.^198^ The dual-modal agents have been utilized for enhanced visualization of tumors, thrombi, stem cells, and nerve injuries, to name a few representative examples.^31,44,194^

Notably, there is a growing interest in the design of dual-modal theranostic probes providing both therapeutic and diagnostic capabilities. PTT is a cancer treatment strategy based on light-absorbing probes converting laser energy into heat to ablate cancer cells with little collateral damage to surrounding healthy tissues. PTT exhibits reduced side effects compared to mainstream cancer treatments like chemotherapy and radiotherapy (RT).^199^ Dual-modal OA–MR image-guided PTT with a single theranostic nanoagent offers complementary advantages for precise lesion localization in the murine brain. Furthermore, dual-modal imaging enables monitoring functional parameters of the tumor environment, such as oxygen and hemoglobin levels in response to therapy, thus allowing continuous adjustment of the intervention. Dual-modal OA–MR contrast agents with high photothermal efficiency under laser radiation have been studied for photothermal applications, including Mn^2+^-doped Prussian blue nanocubes,^195^ MoS_2_/Fe_3_O_4_ NPs,^190^ copper manganese sulfide nanoplates,^187^ mesoporous silica NPs hybridized with manganese dioxide NPs,^200^ and FeSe_2_/Bi_2_Se_3_ nanosheets.^201^ Recently, magnetic responsive immunostimulatory nanoagents (MINPs) were explored for OA–MR image guided PTT on primary tumors and hyperthermia-triggered immunotherapy on distant metastatic tumors. The MINPs were fabricated using a double-emulsion process and involved three key components: (1) SPIO NPs with combined MR, OA, and PTT functionality, (2) cytosine–phosphate–guanine (CpG) oligodeoxynucleotides (ODNs), immunoadjuvants increasing antitumor immunity, (3) monomethoxypoly(ethylene glycol)-poly(lactic-*co*-glycolic acid)-polyl-lysine (mPEG-PLGA-PLL) triblock copolymers, which are cationic carriers to encapsulate the aforementioned agents (Fig. 9(g)). MINPs displayed broad absorption spectra, featuring a peak at 262 nm, which is indicative of the characteristic absorption peak of CpG ODNs (Fig. 9(h)). Upon intravenous injection of MINPs and subsequent magnetic targeting with external magnetic field exposure, a rapid increase in OA signals and decrease in *T*_2_-weighted signals were observed and sustained for 24 h demonstrating the accumulation and retention of the MINPs at the tumor site^41^ (Fig. 9(i)).

## Multimodal combinations with ionizing imaging techniques

Nuclear medicine imaging methods, such as positron emission tomography (PET) or single-photon-emission computed tomography (SPECT), are molecular imaging techniques that map the biodistribution of extrinsically administered radioactive tracers to provide information on organ function and cellular (metabolic) activity at the whole-body level.^202,203^ Due to their poor anatomical contrast and spatial resolution, these techniques are commonly combined with X-ray computed tomography (CT) that provides the missing anatomical context with high spatial resolution but suffers from low molecular sensitivity.^204^ Combination between OA and ionizing imaging modalities brings together the functional OA readouts related to blood oxygenation, the 3D whole-body structural information from CT, and other valuable functional and molecular information from PET or SPECT. Considering the widespread use of PET–CT in clinical oncology, OA can further impact clinical cancer diagnosis and treatment monitoring by providing additional information on tumor hypoxia. To this end, such multimodal combinations have been performed with independent measurements from stand-alone scanners.

### OA and X-ray CT

**Fig. 10:**
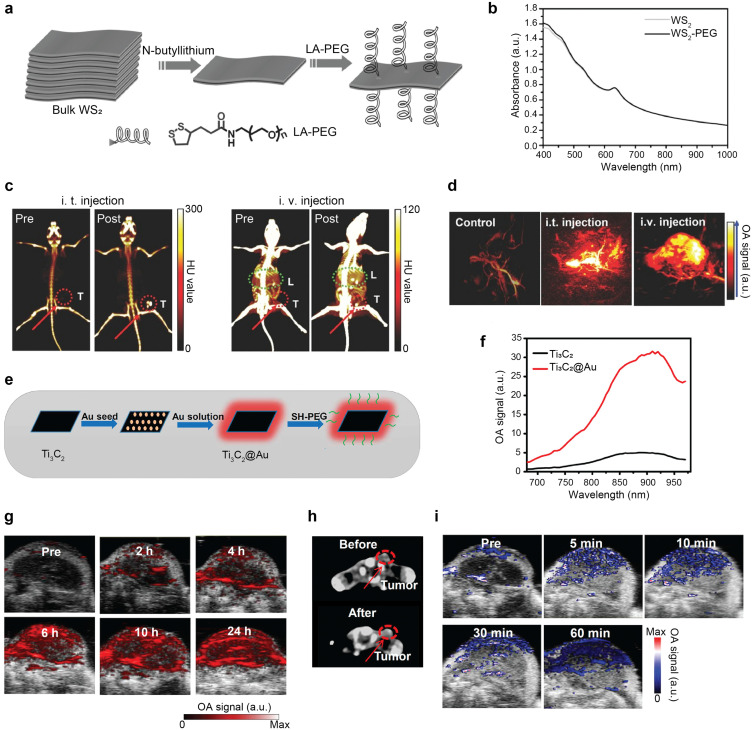
OA–CT dual-modal imaging. (a) Synthesis of WS_2_-PEG nanosheets. (b) Optical absorption spectra of WS_2_ and WS_2_-PEG. (c) CT images of mice pre- and post-intratumoral (i.t.) and intravenous (i.v.) injection of WS_2_-PEG. Red dashed circle indicates the tumor and green dashed circle indicates the mouse liver. (d) OA images of murine tumors pre- and post-i.t. or i.v. injection of WS_2_-PEG. Reprinted with permission from ref. 207. Copyright 2013 John Wiley and Sons. (e) Synthesis of Ti_3_C_2_@Au. (f) OA signal intensities in the NIR window for Ti_3_C_2_ and Ti_3_C_2_@Au. (g) OA images of tumor-bearing mice at different time intervals following injection of Ti_3_C_2_@Au. (h) CT images of mice pre- and postinjection of Ti_3_C_2_@Au. Red dashed circle indicates the tumor location. (i) Blood oxygen saturation images pre- and post-radiation at different time intervals. Reprinted with permission from ref. 208. Copyright 2019 American Chemical Society.

CT contrast stems from the interaction (attenuation) of X-rays and inner-shell electrons. Thereby, probes containing elements with a high atomic number (commonly iodine or barium) are frequently used as CT contrast agents in clinical settings.^205^ Diverse dual-modal OA–CT diagnostic and theranostic agents have been extensively explored (Table 6). In all cases, multimodal imaging has been realized sequentially with preclinical OA and CT scanners. PEGylated tungsten disulfide nanosheets (WS_2_-PEG) have been reported as dual-modal OA–CT contrast agents. The synthesis procedure involves fabrication of single-layered WS_2_ nanosheets from bulk WS_2_ by means of the Morrison method^206^ and a subsequent surface coating with lipoic acid conjugated PEG (Fig. 10(a)). The probe was shown to provide high NIR-absorbance (Fig. 10(b)) and high X-ray attenuation.^207^*In vivo* CT imaging of mice bearing 4T1 breast tumors before and after intratumoral injection of WS_2_-PEG revealed an almost 6-fold enhancement of the CT signal at the tumor site (Fig. 10(c)), while OA signals were enhanced by approximately 5 and 3 times following intratumoral and intravenous injection, respectively (Fig. 10(d)). Hybrid OA–CT imaging of WS_2_-PEG exploited the complementary strength of both modalities. Whole-body imaging achieved with CT was complemented with the high spatial resolution images of tumor microstructures acquired with OA. In a recent study, titanium carbide embedded in gold nanocomposites (Ti_3_C_2_@Au) were developed to study photothermally-enhanced RT in the NIR-II window.^208^ The nanocomposites, synthesized by Au growth on the surface of the Ti_3_C_2_ nanosheets (Fig. 10(e)), displayed high optical absorption for both the NIR-I and the NIR-II windows (Fig. 10(f)). Following intravenous injection of Ti_3_C_2_@Au to 4T1 tumor bearing mice, enhanced OA signal with maximum at 24 h post-injection was observed at the tumor site, demonstrating the high uptake of the probe due to the EPR effect (Fig. 10(g)). Similarly, increased CT signals 24 h after injection corroborated the OA imaging results (Fig. 10(h)). Blood oxygen saturation measured with OA doubled that of preinjection levels and was maintained for an hour post the NIR-II irradiation (Fig. 10(i)), indicating that the mild photothermal heating could effectively improve the tumor hypoxia microenvironment thus enhance cancer RT performance.^208^

**Table 6:** OA–CT, OA–SPECT, and OA–PET contrast materials

Modality	Category	Subcategory	Contrast agents	Absorption	Application (nm)	Ref.
OA–CT	Inorganic materials	Nobel metals	Pd@Au-PEG	700	PTT of breast cancer model	209
		Semimetals	Bi_2_Se_3_ NSS	400–900	HeLa tumor, PTT, Chemo	210
			Bi-LyP-1 NPs	200–1100	PTT/RT of breast cancer model	48
		Transition-metal dichalcogenides	WS_2_-PEG	700–1000	PTT of breast cancer model	207
		Multimetallic particles	Ti_3_C_2_@Au	700–1000	PTT/RT of breast cancer model	208
	Hybrid materials	Metal/semimetals-polymer	TaO_*x*_@PPy NPs	300–900	PTT of glioma	211
			Bi@ PPy-PEG NHs	400–900	PTT of breast cancer model	212
		Other	Au@PB NPs	650–900	PTT of colorectal adenocarcinoma	213
OA–SPECT	Organic materials	Small molecule	A1094@RGD-HBc	1094	Brain gliomas	214
			Cy@Silk-^99m^Tc	600–900	PTT of orthotopic osteosarcoma	215
			^125^I-MB	667	SLN mapping	47
	Hybrid materials		CPMSN@^125^I-SD	680–860	Brain ischemia treatment	216
OA–PET	Inorganic materials	Metal sulfide	^64^CuS-NPs	1064	SLN mapping	53
		Other	^89^Zr-bGNR@MSN(DOX)-PEG	805	Chemo-PTT therapy of breast cancer model	51

### OA and SPECT imaging

**Fig. 11:**
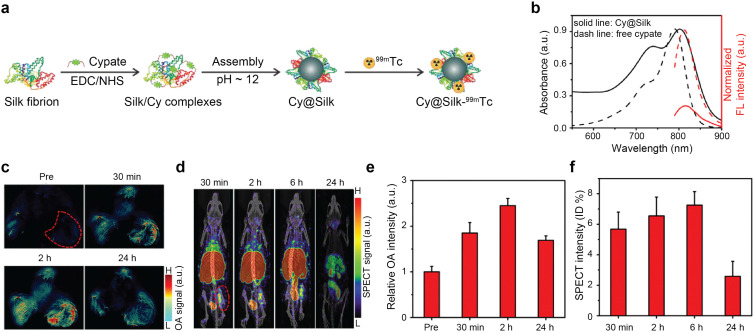
OA–SPECT dual-modal imaging. (a) Synthesis of Cy@Silk-^99m^Tc nanoagents. (b) Optical absorption and FL spectra of Cy@Silk and free cypate. (c) *In vivo* dynamic OA imaging of osteosarcoma-bearing mice pre- and post-injection of Cy@Silk. Tumor region is indicated by the red dashed area. (d) *In vivo* dynamic SPECT imaging of osteosarcoma-bearing mice at 30 min and 2, 6, and 24 h post-injection of Cy@Silk-^99m^Tc. Tumor region is indicated by the red dashed area. (e)–(f) OA and SPECT signal intensities at the tumor site at different time points. Reprinted with permission from ref. 49. Copyright 2019 American Chemical Society.

Nanoagents labeled with radionuclides, *e.g.*^125^I,^47,216^^131^I,^214^ and ^99m^Tc^41^ have been further studied for OA–SPECT hybrid imaging. OA–SPECT–CT probes have been used as diagnostics agents for sentinel lymph node mapping^47^ and tumor imaging,^214,217^ and substantially as theranostics agents to perform image-guided PTT,^209–213,218,219^ combined PTT and RT,^48,208,220,221^ and combined PTT and chemotherapy.^222^ A cypate-induced silk nanoagent labeled with ^99m^Tc was used for dual-modal OA-SPECT imaging and theranostic applications^49^ The synthesis procedure consisted in chemically linking the cypate molecule to the amine groups of silk fibroin and subsequent formation of NPs (Cy@Silk) through self-assembly under alkaline conditions (Fig. 11(a)), which provided strong NIR absorption (Fig. 11(b)). *In vivo* dynamic OA-SPECT imaging of mice bearing osteosarcomas was performed *via* intravenous injection of the radionuclide labeled NP Cy@Silk-^99m^Tc (Fig. 11(c) and (d)). OA signals were increased by 4.3-fold at the tumor location 2 h post-injection, demonstrating the accumulation of NPs *via* the EPR effect (Fig. 11(c) and (e)). Similarly, SPECT imaging was realized at 30 min, 2, 6, and 24 h after injection of Cy@Silk-^99m^Tc, and a high uptake was observed at the tumor location and through the removal pathway of the nanoagent (Fig. 11(d) and (f)). The OA–SPECT–CT contrast materials are further summarized in Tables 6 and 7.

**Table 7:** Multimodal OA contrast materials

Modality	Category	Subcategory	Contrast agents	Absorption (nm)	Application	Ref.
OA–MR-Raman	Hybrid materials		MPR nanoparticle	540	Brain tumor resection	31
OA–MR–CT	Inorganic materials	Multimetallic composites	MnO@Au NCs	400–1100	Liver cancer model	197
			MnO_2_-mSiO_2_@Au NPs	680–1064	PTT/RT in breast cancer model	200
		Other	MPDA-WS_2_@MnO_2_	300–808	PTT/RT in breast cancer model	221
			Gd_2_O_3_/BSA@MoS_2_-HA NPs	200–900	PTT/RT in breast cancer model	220
	Hybrid materials		Au@MIL-88(Fe)	720	Glioma	217
			Gd-PEG-Bi NPs	700 –900	PTT of glioma	219
OA–MR–PET	Inorganic materials		^64^Cu-MoS_2_-IO-(d)PEG	400–1000	PTT in breast cancer model	223
	Hybrid materials	Melanin-based particles	^64^Cu-Fe-RGD-PEG-MNP	300–1000	Glioblastoma	196
			^64^ Cu-MMNs	500–1100	PTT of glioma, γ-irradiation protection	224
			^64^Cu-AMF	N.A.	Colon cancer, liver cancer	225
		Other	CDPGM NPs	500–900	Chemo-PTT therapy of glioma	226
			^64^Cu-NOTA-RGO-IONP-^1st^PEG-^2nd^PEG	700–1000	Breast cancer model	227
OA–MR–US	Inorganic materials	Iron particles	PBNCs	750	MSC labeling	179
OA–CT–FL	Organic materials		PFOB@IR825-HA-Cy5.5 NPs	870	PTT of colon cancer	218
			Pdots-DOX-iohexol@hydroge	800	Chemo-PTT therapy in breast cancer model	222
OA–PET–FL	Organic materials	Small molecule	[^18^F]CDA-3	798	Aβ plaque imaging in AD	50
		Other	Pheo ss-InFroMs	∼660	Intestine	228
OA–MR–CT–PET	Inorganic materials	Multimetallic composite	^64^Cu-FeSe_2_/Bi_2_Se_3_-PEG	700–1000	PTT/RT in breast cancer model	201

### OA–PET combinations

The PET technique is widely used in disease diagnosis, monitoring responses to therapy, and pharmacokinetic studies. It allows whole-body quantitative tracking of radionuclide-labeled tracers with high sensitivity, but suffers from limited spatial resolution, slow imaging speed, absence of anatomical information, and a need for radionuclide production facility.^203^ The low spatial resolution of preclinical PET scanners relative to the size of internal structures in small animals often leads to partial volume effects.^229^ The higher resolution of OA imaging can help to better exploit the excellent sensitivity and deep tissue quantitative imaging capacity of PET. OA provides unique oxygenation readouts that can be exploited in a dual-modal OA–PET combination, which has been used *e.g.* for image-guided cancer therapy, gut imaging, and neuroimaging of amyloid-beta plaque.^50,51,224,226,228,230^

**Fig. 12:**
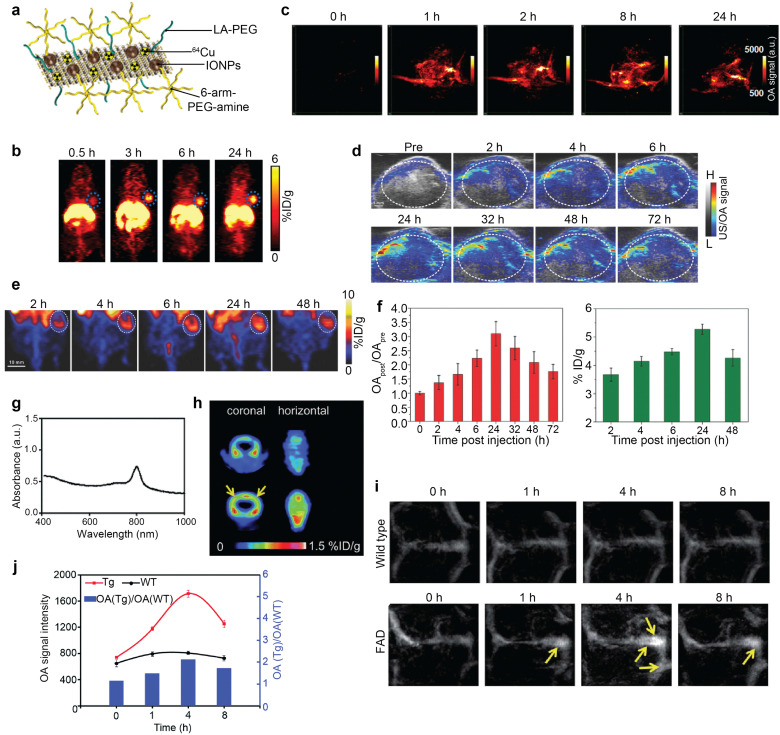
OA–PET dual-modal imaging. (a) Schematic presentation of ^64^Cu-MoS_2_IO-(d)PEG. (b) PET images of mice pre- and post-intravenous injection of ^64^Cu-MoS_2_IO-(d)PEG. Blue dashed circle indicates the tumor site. (c) OA images of tumor pre- and post-injection of polydopamine–gadolinium–metallofullerene (PGM) NPs. Reprinted with permission from ref. 223. Copyright 2015 American Chemical Society. (d) OA images of U87MG-tumor-bearing mice pre- and postinjection of PGM NPs. (e) PET images of U87MG tumor-bearing mice at various time points post injection of core–satellite polydopamine–gadolinium–metallofullerene (CPGM) NPs. (f) OA signal intensity changes and time-dependent tumor uptake of CPGM NPs. Reprinted with permission from ref. 226. Copyright 2017 John Wiley and Sons. (g) Absorption spectrum of [^18^F]CDA-3. (h) [^18^F]CDA-3 PET images of wildtype (WT) and transgenic (Tg) mice. (i) OA images of Tg and WT mice postinjection of [^18^F]CDA-3 at different time points. (j) OA signal intensity changes of Tg and WT mice following [^18^F]CDA-3 injection (left axis) and the OA(Tg)/OA(WT) ratio at different time points (right axis). Reprinted with permission from ref. 50. Copyright 2017 Royal Society of Chemistry.

Positron-emitting radioisotopes, such as ^64^Cu^53,201,223–225,227,230^ and ^18^F,^50^ have been combined with OA nanoprobes to establish the dual contrast (Tables 6 and 7). IONPs self-assembled on MoS_2_ nanosheets labeled with ^64^Cu (^64^Cu-MoS_2_-IO-(d)PEG) (Fig. 12(a)) act as a biocompatible triple-modality OA–PET–MR contrast agent.^223^ This probe exhibits high NIR-absorbance attributed to MoS_2_ nanosheets and allowed imaging the distribution and pharmacokinetics of the ^64^Cu isotope with PET in an animal tumor model (Fig. 12(b)). Similarly, elevated OA signal levels were observed following administration of the probe peaking at ∼8 h post injection, thus corroborating tumor retention of the probe (Fig. 12(c)). The whole-body imaging capacity and high sensitivity of PET, combined with the high-resolution functional OA readings, were used for image-guided PTT with 808 nm laser irradiation (0.78 W cm^−2^, 5 min), resulting in complete tumor ablation. Another notable multimodal nanotheranostic probe is ^64^Cu-labeled doxorubicin (DOX)-loaded polydopamine (PDA)-gadolinium-metallofullerene (CDPGM), which was used for OA–PET–MR imaging-guided chemo-photothermal combination therapy.^226^ The PDA core of the CDPGM acts as an OA contrast agent with high NIR-absorption and photothermal stability. *In vivo* imaging of U87MG-tumor-bearing mice following intravenous injection of the probe manifested gradually enhanced OA contrast at the tumor site (Fig. 12(d)). The uptake efficiency of the probe within the tumor was measured quantitatively with PET, indicating increased uptake at 24 h postinjection followed by a subsequent decrease (Fig. 12(e) and (f)). OA signal intensity showed a maximum 3-fold increase 24 h post-injection and decreased at later time points in accordance with the PET results (Fig. 12(f)).

Dual-modal neuroimaging of disease models may enable validating the functional OA readouts with a well-established PET modality. Also, the high spatial resolution and molecular information from OA can enhance the capabilities of PET for accurate diagnosis and localization of lesions. Recent work incorporated a ^18^F labeled croconium dye in an amyloid probe ([^18^F]-CDA-3) to visualize amyloid beta (Aβ) deposits in the Alzheimer's disease (AD) murine brain.^50^ The designed OA–PET–FL probe exhibited strong NIR-absorption (Fig. 12(g)) and photothermal efficiency, displaying distinct distribution pattern in transgenic as opposed to wildtype mice (Fig. 12(h)). Time-lapse OA images displayed enhanced signals in cerebral vessels in the brains of transgenic mice predominantly in the sagittal sinus, while the signal intensity remained almost identical in wildtype mice (Fig. 12(j) and (i)). The OA signal intensity ratio between transgenic and wildtype mice peaked at 4 h following injection, suggesting gradual accumulation in the vessels and high binding to the Aβ plaque.

## Conclusions and outlook

Bioimaging techniques have become essential tools in basic and translational research.^231^ In parallel, contrast materials are being developed for improved visibility of specific tissues, cells, physiological processes, or molecular pathways in health and disease.^232^ Each modality comes with specific strengths and weaknesses pertaining to its costs, portability, spatial and temporal resolution performance, penetration depth, or molecular sensitivity and specificity. A common approach to achieve comprehensive examinations is to integrate different modalities into hybrid (multimodal) imaging systems providing complementary information. The added value of multimodal imaging is manifested *e.g.* with the widespread use of PET–CT scanners in oncology, brain diseases and other fields, where functional (metabolic) information retrieved with PET is fused onto CT anatomical images.^233^ Proper selection of the contrast agents is essential for attaining optimal multimodal imaging performance. Multiple substances can be administered, *e.g.* to facilitate co-registration of multimodal images by enhancing angiographic contrast.^234^ However, multimodal agents with suitable pharmacokinetic properties, which simultaneously provide detectable contrast in all the employed modalities, are generally preferred.

As a relatively new addition to the bioimaging arsenal, OA techniques are experiencing rapid technological progress. Initially, light absorption properties of the common optical contrast agents have been exploited for contrast-enhanced OA imaging.^235^ More recently, the unique advantages of OA imaging are fostering the development of dedicated contrast materials with physical, chemical, and biochemical characteristics tailored to specific applications.^18,232,235,236^ Dissemination of OA imaging systems among biomedical researchers helps crystallizing the key advantages and limitations of this modality with respect to alternative imaging methods, thus reinforcing the need for developing multimodal OA imaging approaches. Here we provide a detailed description of the benefits and challenges associated with state-of-the-art multi-modality systems, contrast materials, and respective applications, including the combinations of OA with US, FL, OCT, Raman, MRI, CT, SPECT, and PET imaging. We delve into specific strengths achieved by merging the information stemming from absorption of photons in biological tissues with complementary imaging contrast mechanisms based on reflection, scattering, and attenuation of US waves, FL emission, proton relaxation, diffusion, and perfusion-weighted contrast in MRI, X-ray attenuation in CT, single-photon or positron-emitting radionuclides in SPECT and PET. Beyond enriching the structural, molecular, functional, and metabolic information retrieved from living tissues, hybridization with other methods can also help solving or mitigating some of the challenges commonly attributed to the OA methods, such as limited imaging depth, image artifacts associated with acoustic heterogeneities, or wavelength-dependent light attenuation adversely affecting the image quantification capacity.

An important advantage of OA imaging is its label-free capacity, *i.e.* the ability to operate based solely on endogenous chromophores such as hemoglobin, lipids, water, collagen, or melanin. The high concentration of hemoglobin in red blood cells enables label-free angiographic imaging as well as monitoring of hemodynamic changes and oxygen saturation, which has provided new insights in neuroscience, oncology, and other fields.^237,238^ However, the strong absorption background by hemoglobin is a double-edged sword that may hamper the detection of other molecules in the presence of blood. Exogenously administered molecules and particulate agents have been used to enhance the performance of stand-alone and multimodal OA imaging.^18,235^ Accumulation of such agents in specific tissues and cells reveals the presence of pathological microenvironments or protein targets. Organic fluorescent dyes are excellent light absorbers thus ideally suited for hybrid OA–FL imaging, while radio-labelled molecules can readily facilitate hybridization with nuclear medicine methods. More flexibility is achieved by capitalizing on the capabilities of modern nanotechnology to produce multiple types of NPs that can improve molecular targeting efficiency or encapsulate multiple contrast substances and/or therapeutic moieties. Nanoparticulate contrast agents are widely used for contrast enhancement in various imaging modalities thus can readily be engineered to exhibit light absorption properties to achieve optimal OA multimodal performance. Larger micrometer-scale particles and microbubbles are commonly used for enhancing US contrast and enabling super-resolution imaging beyond the acoustic diffraction barrier.^239^ Recently, microparticles have also been used to realize similar super-resolution approaches in FL and OA imaging.^240–242^ Further developments in multimodal contrast materials should not only focus on improving their efficiency of signal generation but also optimizing the biodistribution properties, targeting capacity, biodegradability, and biocompatibility to reduce toxicity and administered doses.

Hybridization of OA with other imaging modalities may face technical challenges. OA imaging traditionally requires an opaque transducer array detector to collect signals over a broad angle surrounding the object thus limiting the physical space for signal collection with other modalities. Furthermore, the OA signal sampling electronics emits electromagnetic radiation, potentially interfering with other imaging modalities, such as MRI, where shielding and proper grounding are essential to minimize electromagnetic interference. Besides, coupling media (*e.g.*, water or gel) is commonly required for efficient detection of the generated OA responses, which may introduce additional compatibility issues and image artifacts when combining OA with other modalities. Multimodal system integration also comes with an inevitable cost of higher complexity in terms of the system's hardware design, synchronization of data acquisition, and image interpretation. Nonetheless, with the advent of more affordable lasers and US detection platforms along with new high-performance contrast materials, multimodal OA imaging is expected to find broad applicability in diverse areas, such as oncology, dermatology, neurology, and cardiovascular diseases.

We have shown that multimodal OA approaches can impact a myriad of biomedical research fields. When combined with MRI or US, the simultaneous multi-parametric hemodynamic readings can significantly enhance neuroscience research into resting-state or stimulus-evoked brain activity with FL imaging assisted with calcium or voltage indicators providing additional insights on the neural activity. Combining MRI or CT for anatomical tumor characterization with the functional and molecular contrast offered by OA can enhance oncological research, where multimodal agents further enable therapeutic interventions, *e.g. via* PTT or PDT. The use of high-frame-rate imaging technologies, such as OA, US, or OCT, is essential in cardiovascular biology, where simultaneous imaging with diverse contrast mechanisms can facilitate the study of electromechanical wave propagation and cardiac arrythmias. Verification and cross-validation of experimental results with other imaging methods have also been essential for the wider adoption of OA imaging approaches. This is of particular relevance for successful clinical translation of OA imaging where the physicians trained with well-established clinical imaging methods may lack the knowledge to interpret OA images. Multimodal imaging systems based on hybridization with routinely used clinical approaches can then help identify OA biomarkers of clinical relevance for diagnostic purposes. Multimodal contrast agents with clear OA signatures can also potentially be used in patients. Light-absorbing organic dyes, such as ICG, Evans blue, or Methylene blue, are approved for clinical use, while contrast-enhanced US, MRI, or CT is also routinely used. This is expected to facilitate the regulatory approval of new hybrid formulations provided the added value of multimodal OA imaging has been demonstrated. All in all, the complementary anatomical, functional, molecular, and metabolic information rendered with multimodal imaging systems facilitates the full exploitation of the unique advantages and complementarity of OA toward a more comprehensive understanding of biological processes.

## Abbreviations

NP: Nanoparticle
PFC: Perfluorocarbon
MB: Methylene blue
BI: Black ink
MSOT: Multi-spectral optoacoustic tomography
AuMBs: Albumin-shelled microbubbles with encapsulated gold nanorods
PAnDs: Plasmonic noble metal nanoparticles-encapsulated PFC nanodroplet
PSMAP/ICG NBs: Prostate-specific membrane antigen-targeting, indocyanine green-loaded nanobubbles
SAPTN: Stimulator of interferon genes agonist-based photoimmunothernostic nanomedicine
MC-PSE: GSH-activatable probe
DTP-DPTQ NPs: Fluorophore with dithienopyrrole as the donor and 6,7-diphenyl-[1,2,5]thiadiazolo [3,4-*g*]quinoxaline as the acceptor
PTT: Photothermal therapy
RT: Radiotherapy
GGT: γ-Glutamyltranspeptidase
LET-12: IR-1064 self-assembly modified with a choline and acetylcholine analogue, 2-methacryloyloxy ethyl phosphorylcholine
CyA: Aminopeptidase N (APN)-activated type I phototheranostic probe
FMP&N-FMP: FAPα-activatable molecular pro-theranostic probes organic materials
P-CyPt: NIR merocyanine fluorophore capped with an ALP-recognition phosphate group (PO_3_H), a GSH-reducible CDDP prodrug (Pt(iv)), and a hydrophobic d-Phe-d-Phe (FF) dipeptide
CTSK-APPA: Hemicyanine dye (CyN_3_OH) caged by a cathepsin K (CTSK)-cleavable peptide sequence on one side and functionalized with osteophilic alendronate
AIEgens: Aggregation-induced emission luminogens
MPNPs: AIEgens MTPE-TT and chemotherapeutic drug PTX conjugated with 4-nitrobenzyl chloroformate
C-NTBD: Aggregation-induced emission luminogens with benzodithiadiazole as the electron acceptor
EMT-NPs: Endoplasmic reticulum and mitochondria dual-targeting nanoparticles
PDX: Patient-derived xenograft
SPCy: Semiconducting polymer conjugated with a hemicyanine (hemi-Cy) dye caged by a NE-cleavable peptide as the side chain
P_2_NPs: Semiconductor polymer P2 encapsulated with amphiphilic PEGylated phospholipid
CF-SPNs: Semiconducting polymer nanoparticles with fluorescence resonance energy transfer (FRET) and chemiluminescence resonance energy transfer
mPPy@COF-Por: Membrane-coated core–shell nanomotor consisting a porphyrin-decorated COF Shell and polypyrrole core
AuNNPs–Ag_2_S Ve: Nanogapped gold nanoparticles (AuNNPs)–silver sulfide (Ag_2_S) vesicle (Ve)
AgIONPs: Silver-iron oxide nanoparticles
mdGC: Gold nanorods with carbon-based nanomaterials
PCD: Permeable carbon dots
V_2_C-TAT@Ex-RGD: Vanadium carbide (V_2_C) QDs PTA modified with TAT peptides and packaged into engineered exosomes (Ex) vector with RGD modification
PhAg NPs: NIR light absorbing Silver NPs loaded with Pheophorbide-a
CFNPs: Croconium dye (Croc)–ferrous ion (Fe^2+^) nanoprobes
CDT: Chemodynamic therapy
Au^0^–Por@FeCO: Gold-based porphyrinic coordination polymer nanosheet loaded with triiron dodecarbonyl
MTCN: Microenvironment-tailored catalytic nanoprobe
UCN: Upconversion nanocrystal
CRUN: Enzyme-responsive cross-linking of rare-earth UCNs
HCy5/Cy7-UCNs: Lanthanide-doped upconversion nanocrystals with ROS- and RNS-sensitive NIR cyanine fluorophores
AHZ NPs: Acid-labile metal–organic frameworks with mineralized hyaluronidase (HAase) and encapsulated Ag_2_S nanodots
HSC-2: Zeolite–carbon-based nanozymes
MSbNSs: Monodispersed mesoporous Sb nanospheres
QDs: Quantum dots
MOF: Metal–organic frameworks
Nanowontons: Zero-valence ferromagnetic cobalt particles with a gold coating
CP-IO: Nanocomposites of conjugated polymers and iron oxide nanoparticles
SPIO@Au nanoparticles: Containing superparamagnetic iron oxide coated with gold
Fe^3+^-PEG-MNP: Polyethylene glycol-modified magnetic nanoparticle
MSC: Mesenchymal stem cells
Gd–Fe/HCSs: Hollow carbon nanospheres dotted with GdPO4 and γ-Fe_2_O_3_ nanoparticles
HA-MnO@MSN: Core–shell nanoparticle consists of manganese oxide and hyaluronic acid-conjugated mesoporous silica nanoparticle
Au-IO NP: Gold/iron oxide multimetallic nanoparticles
DDNPs: Dual-modality and dual-ligand nanoparticles
PB: Prussian blue
MSIOs: MoS_2_/Fe_3_O_4_ composite
MINPs: Composites of superparamagnetic iron oxide nanoparticles and cytosine–phosphate–guanine oligodeoxynucleotides
NSS: Nanoscale spherical sponges
Bi@PPy-PEG: NHs polyethylene glycol-modified polypyrrole-coated bismuth nanohybrids
Bi-LyP-1: NPs peptide (LyP-1)-labeled ultrasmall semimetal bismuth nanoparticles
CPMSN@^125^I-SD: Cobalt protoporphyrin IX-loaded mesoporous silica nanoparticle with a 125I conjugated/spermine-modified dextran polymer
A1094@RGD-HBc: Mesoionic dye A1094 encapsulated in Arg-Gly-Asp-modified hepatitis B virus core protein (RGD-HBc)
Cy@Silk-^99m^Tc: Indocyanine green analogue cypate-induced silk fibroin self-assembly nanoagents
SLN: Sentinel lymph node
MPR nanoparticle: Gold–silica–based SERS nanoparticle coated with Gd^3+^ ions
MPDA: Mesoporous polydopamine nanosponges
Gd-PEG-Bi NPs: Pure Bi NPs conjugated with gadolinium-diethylenetriaminepentaacetic acid-bis-tetradecylamide
MNP: Melanin nanoparticle
PGM: Polydopamine–gadolinium–metallofullerene
CPGM: Core–satellite polydopamine–gadolinium–metallofullerene
CDPGM: Radionuclide-^64^Cu-labeled doxorubicin-loaded polydopamine–gadolinium–metallofullerene core–satellite nanotheranostic agent
^64^Cu-MMNs: ^64^Cu-labeled magnetic melanin nanoparticles
PBNCs: Prussian blue nanocubes
Aβ: Amyloid beta
AD: Alzheimer's disease
MINPs: Magnetic responsive immunostimulatory nanoagents
CpG: Cytosine–phosphate–guanine
ODNs: Oligodeoxynucleotides
FOV: Field-of-view
SAN: Stacked gold nanodisk
TUT: Transparent ultrasound transducer
EPR: Enhanced permeability and retention

## Author contributions

ZC and DR outlined the review content and structure. QZ, LT, ZC, IG, and XLDB drafted the individual sections. DR supervised the work and edited the manuscript. All authors contributed constructively to the manuscript writing and revisions.

## Conflicts of interest

The authors declare no competing interests.
